# AIP1 is a novel Agenet/Tudor domain protein from Arabidopsis that interacts with regulators of DNA replication, transcription and chromatin remodeling

**DOI:** 10.1186/s12870-015-0641-z

**Published:** 2015-11-04

**Authors:** Juliana Nogueira Brasil, Luiz Mors Cabral, Nubia B. Eloy, Luiza M. F. Primo, Ito Liberato Barroso-Neto, Letícia P. Perdigão Grangeiro, Nathalie Gonzalez, Dirk Inzé, Paulo C. G. Ferreira, Adriana S. Hemerly

**Affiliations:** Instituto de Bioquímica Médica Leopoldo de Meis, Universidade Federal do Rio de Janeiro, Rio de Janeiro, Brazil; Departamento de Biologia Celular e Molecular, Universidade Federal Fluminense, Niterói, Rio de Janeiro Brazil; Department of Plant Systems Biology, Flanders Institute for Biotechnology (VIB), Ghent, Belgium; Programa de Biologia Celular, Instituto Nacional de Câncer, Rio de Janeiro, Rio de Janeiro, Brazil; Departamento de Química, Universidade Federal do Ceará, Fortaleza, Ceará Brazil

**Keywords:** Agenet/Tudor, Tudor, DUF7, DUF724, ABAP1, Chromatin remodeling, Cell cycle, Arabidopsis

## Abstract

**Background:**

DNA replication and transcription are dynamic processes regulating plant development that are dependent on the chromatin accessibility. Proteins belonging to the Agenet/Tudor domain family are known as histone modification “readers” and classified as chromatin remodeling proteins. Histone modifications and chromatin remodeling have profound effects on gene expression as well as on DNA replication, but how these processes are integrated has not been completely elucidated. It is clear that members of the Agenet/Tudor family are important regulators of development playing roles not well known in plants.

**Methods:**

Bioinformatics and phylogenetic analyses of the Agenet/Tudor Family domain in the plant kingdom were carried out with sequences from available complete genomes databases. 3D structure predictions of Agenet/Tudor domains were calculated by I-TASSER server. Protein interactions were tested in two-hybrid, GST pulldown, semi-in vivo pulldown and Tandem Affinity Purification assays. Gene function was studied in a T-DNA insertion GABI-line.

**Results:**

In the present work we analyzed the family of Agenet/Tudor domain proteins in the plant kingdom and we mapped the organization of this family throughout plant evolution. Furthermore, we characterized a member from *Arabidopsis thaliana* named AIP1 that harbors Agenet/Tudor and DUF724 domains. AIP1 interacts with ABAP1, a plant regulator of DNA replication licensing and gene transcription, with a plant histone modification “reader” (LHP1) and with non modified histones. *AIP1* is expressed in reproductive tissues and its down-regulation delays flower development timing. Also, expression of ABAP1 and LHP1 target genes were repressed in flower buds of plants with reduced levels of AIP1.

**Conclusions:**

AIP1 is a novel Agenet/Tudor domain protein in plants that could act as a link between DNA replication, transcription and chromatin remodeling during flower development.

**Electronic supplementary material:**

The online version of this article (doi:10.1186/s12870-015-0641-z) contains supplementary material, which is available to authorized users.

## Background

Chromatin is a highly regulated and dynamic structure that is constantly remodeled during development in order to couple gene transcription events with cellular processes such as cell division and differentiation. Histone modifications are an important mechanism regulating chromatin remodeling, and they are carried out by specific enzymes followed by recognition by so-called “histone reader” proteins [1]. The Agenet and Tudor domains, together with the Chromatin-binding (Chromo), Bromo, Bromo-Adjacent Homology (BAH), PWWP (conserved Proline and Tryptophan) and Malignant Brain Tumor (MBT) domains are known as histone modification “readers” and present in many proteins classified as chromatin remodelers [2, 3]. The Agenet domain was first described as a plant-specific member of the larger Royal domain family because of its similarity with animal Tudor domain from Fragile X Mental Retardation Protein (FMRP) [3]. Afterwards, the occurrence of Agenet domain was also reported in human proteins [4, 5], therefore this protein family is now referred as Agenet/Tudor domain family. In the last years, more insights on how Agenet/Tudor proteins function are being revealed [5–9], including the identification of an RNA-binding domain (KH) in the neighborhood of the Agenet/Tudor domains from human FMRP, that is responsible for the RNA-binding function [5]. Still, very little is known about the role of Agenet/Tudor domain in plants and it’s importance for plant development.

Agenet/Tudor domain proteins are widespread in the plant kingdom, and 28 genes were identified in the *Arabidopsis thaliana* genome [2]. EMSY-like N-Terminal (ENT), BAH, Plant Homeodomain (PHD) and DUF724 domains are reported to often co-occur with plant Agenet/Tudor domains, possibly conferring diverse functions to these proteins [3]. Plant ENT domains resemble those of the human oncoprotein EMSY, reported as repressors of the transcriptional activator function of the tumor suppressor BRCA2 [2]. The BAH domain is involved in epigenetic regulations acting in the formation of an aromatic cage that binds histone H3 lysine 9 dimethylation (H3K9Me2) of nucleosomes, interplaying DNA methylation and histone modification [10]. PHD domains are a class of Zinc Finger (ZnF) motif that promotes protein-protein interactions in multi-protein complexes and participates in chromatin remodeling and ubiquitination processes [11]. DUF724 domain was reported to be involved in mediating protein-protein interaction [4]. So far, only Agenet/Tudor that also contains ENT domain have been functionally characterized in plants. In Arabidopsis, AtEMSY-like 1 (AtEML1) and AtEMSY-like 2 (AtEML2) have been described to interact with the transcription factor Enhanced Downy Mildew 2 (EDM2) responsible for repressing expression of the *Flowering Locus C* (*FLC*), with consequences in flowering time control [12]. Another ENT/Agenet/Tudor protein was reported in maize, named R Interacting Factor1 (RIF 1), that is part of a complex that anchors in chromatin of promoter regions increasing acetylation of Histone 3 Lisyne 9 (H3K9/K14ac), to activate expression of selected genes involved in anthocyanin biosynthesis pathway [13]. In addition, the Arabidopsis Coilin protein, that harbors a C-terminal Agenet/Tudor-like structure without any other classified domain, is able to bind RNA in a non-specific manner with subsequent multimerization, which possibly facilitates its function as a scaffolding protein [14].

Histone modifications have profound effects on gene expression as well as on DNA replication, but it has not been completely elucidated how these processes are integrated. In animals, Agenet/Tudor domain proteins have already been reported to have a role in chromatin modifications during DNA repair, connecting it with cell cycle checkpoints. The tandem Tudor domain containing the tumor suppressor p53 Binding Protein 1 (53BP1) can bind to histone modification that marks double stranded DNA breaks (DSB) [7], as well as interact with methylated RETINOBLASTOMA (RB); in this way, it connects the cell cycle control of RB with DNA damage responses and chromatin remodeling processes [7]. Spindilin is a Tudor domain protein from humans that binds to methylated histone [15], and is also known to bind to mitotic spindle and to respond to DSB [16]. The Tudor domain FMRP has already been implicated in participating in DNA repair by specifically binding to methylated histone that marks DNA damage in human cells during replication stress [6]. In addition, the UHFR1 protein (Ubiquitin-like, containing PHD and RING finger domains 1), also known as ICBP90 in humans, is a Tudor containing domain that has a central role in interconnecting the processes of histone methylation, DNA methylation, DNA repair and cell cycle regulation [9]. UHFR1 is a member of E3 ligase family with RING domain that recruits DNA metyltransferase, and regulates expression of genes important at G1 to S transition phase including RB [9].

In plants, the Armadillo BTB Arabidopsis Protein 1 (ABAP1) was described as a plant regulatory protein that is involved in the control of gene expression and DNA replication [17]. ABAP1 associates with members of the Pre-Replication Complex (pre-RC), and also binds to transcription factors to negatively regulate the transcription of essential pre-RC genes [17]. It participates in a signaling network that controls cell cycle progression from G1 to S phase, by integrating plant developmental signals with DNA replication and transcription controls [17]. DNA replication and transcription are dynamic processes dependent on the chromatin accessibility. Still little is known on the role of histone modifications in coordinating replication and transcription, and how they are integrated with development.

Here we report the identification and characterization of a novel Agenet/Tudor/DUF724 domain protein that interacts with ABAP1, named ABAP1 Interacting Protein 1 (AIP1). First, a general bioinformatics and phylogenetic analyses of the Agenet/Tudor Family domain in the plant kingdom were carried out. It suggests that this family has a third structure conserved in animals and plants. Also, a search in complete plant genomes has shown that Agenet/Tudor have expanded with plant evolution. Thirty members of this family were identified in Arabidopsis and they could be classified in four groups by phylogeny. The expression pattern of the different family members have reveled notorious incidence in reproductive tissues. The Arabidopsis Agenet/Tudor domain protein AIP1 was previously reported as a DUF724 domain protein named DUF7 [6], and will be denoted in this article as AIP1. Besides the interaction with ABAP1, a negative regulator of DNA replication and transcription, here we have identified that AIP1 interacts in vivo with the plant histone modification “reader” LHP1 and with non-modified histones. *AIP1* is expressed in reproductive tissues and its down-regulation delays flower development timing. mRNA levels of ABAP1 and LHP1 target genes were down regulated in flower buds of plants with reduced levels of AIP1. This is the first plant protein harboring Agenet/Tudor and DUF724 domains, which is functionally characterized. The data may suggest that AIP1 could act as a link between DNA replication, transcription and chromatin remodeling during flower development.

## Methods

### *In silico* analyses of proteins containing Agenet/Tudor domain

Agenet/Tudor family proteins were searched by TBLASTN using the following databases: Phytozome [18], the National Center for Biotechnology Information (NCBI) database [19], The Arabidopsis Information Resource (TAIR) database [20] and Congenie databases [21]. The part-length (Agenet/Tudor domain) sequence of *At2g17950* (FSSGTVVEVSSDEEGFQGCWFAAKVVEPVGEDKFLVEYRDLREKDGIEPLKEETDFLHIRPPPPR) was used as a query sequence for TBLASTN. The *e-*value of all the sequences selected was below 1*e* − 5. The presence of conserved domains in all the sequences was checked using the Pfam [22], the SMART [23] and the NCBI databases [19] with e-value below 1*e* − 3.

Multiple sequence alignments were carried out by using MUSCLE 3.6 (http://www.ebi.ac.uk/Tools/msa/muscle/) with the default parameter setting. A phylogenetic tree using neighbor joining method was constructed with the sequences of the members of the Agenet/Tudor protein family aligned by MEGA (version 3.0;) [24]. NJ analyses were done using the following parameters: poisson correction methods, pairwise deletion of gaps, and bootstrap (1000 replicates; random seed). For Domain assiniture we used WebLogo (Web-based sequence logo generating application; Weblogo.berkeley.edu) [25]. See Additional file 13 for sequences used to build Agenet/Tudor signature in plant via WebLogo.

The *in silico* analysis to find a peptide signal of cellular localization in AIP1 amino acid sequence was performed using iPSORT on line software according to [26].

### Protein structural modeling

Structural modeling and visualization of Agenet/Tudor domains were performed using the I-TASSER server for protein 3D structure prediction [27]. The three models generated were visualized and handled using the PyMol package [28]. The structures of the Agenet/Tudor domain were aligned using PyMol, and their primary multiple sequence alignments were calculated using Multalin server [29]. The alignment image with the secondary structure of the most significant model adjusted in it was produced using ESPript [30]. PDBeFOLD [31] was used to evaluate the folding of the Agenet domains and to identify structural homologies in the PDB. The likely function of proteins was predicted using ProFunc [32].

### Plant material and expression analyses

Arabidopsis plants were grown on agar plates or soil under long-day conditions (16 h of light, 8 h of darkness) at 23 °C under standard greenhouse conditions. All analyses *in planta* were performed using the Arabidopsis accession Columbia-0 background. Expression analyses using qRT-PCR are described in Additional file 13. Primers sequences can be found in Additional file 12.

### Analysis of 35S::RFP-AIP1 and 35S::GFP-ABAP1

Transient expression in *Nicotiana benthamiana* for subcellular localization was performed according to [33]. Briefly, plasmids were introduced into *A. tumefaciens* (GV3101). Bacteria cultures grown overnight were centrifuged and pellets were resuspended in 10 mMMgCl2 to an optical density of 0.5 at 600 nm and induced with 200 mM acetosyringone. Leaves of 4–5 week old *N. benthamiana* plants were co-infiltrated with an equimolar bacterial suspension of the two constructs to be tested. Confocal laser scanning images of protein co-localization were recorded 2 days post-infiltration (LSM-700, Carl Zeiss).

### Yeast two-hybrid assay

Yeast two-hybrid assays were carried out according to [17]. Briefly, S*accharomyces cerevisiae* PJ694 strain was co-transformed with 1 μg of the constructs by the Polyethylene glycol/LiAc method and plated on synthetic dropout media without either leucine/tryptophan (-leu/-trp) (to test transformation efficiency); or leucine, tryptophan, and histidine (-leu/-trp/-his) (low stringent condition); or leucine, tryptophan, histidine, and adenine (-leu/-trp/-his/-ade) (high stringent condition), and incubated for 3 days at 30 °C.

### In vitro and semi-in vivo protein interaction assays

AIP1-GST, ABAP1–HIS, ARIA-HIS and LHP1-HIS were produced in cells of *Escherichia coli* strain BL21 (Additional file 13). In vitro GST pulldown analyses were carried out according to [34]. Plant protein extracts and protein gel blots were carried out by standard techniques, according to protocols described in the Additional file 13. Semi-in vivo GST pulldown is described in Additional file 13.

### Tandem Affinity Purification (TAP)

AIP1 CDS was cloned for N-terminal fusion to the TAP tag system under the control of the constitutive cauliflower mosaic virus 35S promoter into the NGSrhino vector. Transformation of Arabidopsis cell suspension cultures were then performed as described in [35]. Tandem affinity purification of protein complexes was done using the protein G and streptavidin binding peptide tag followed by protein precipitation and separation, according to [36]. The protocols of proteolysis and peptide isolation, acquisition of mass spectra by a 4800 Proteomics Analyzer (Applied Biosystems), and MSbased protein homology identification based on The Arabidopsis Information Resource 8.0 genomic database were performed according to [37]. Experimental background proteins were subtracted based on approximately 40 TAP experiments on wild-type cultures and cultures expressing the TAP tagged mock proteins Beta-glucuronidase, red fluorescent protein, and green fluorescent protein [38].

### Analyses of AIP1 mutant plants

T-DNA insertion lines of GABI_645B06 (https://www.gabi-kat.de/) were identified by genotyping using PCR with specific primers for GABI T-DNA insertion and for *AIP1*. For details on molecular and phenotypic analysis of AIP1 mutants see Additional file 13.

## Results

### Agenet/Tudor family members have expanded with the evolution of plants

Most proteins containing Agenet/Tudor domain are still poorly characterized in plants. In order to get more insights into the evolution and possible biological role of these proteins, an *in silico* analysis of the Agenet/Tudor domain in the plant kingdom was performed. To search for proteins belonging to Agenet/Tudor domain family in plants, we used an Agenet/Tudor sequence from the gene *At1g09320* to perform TBLASTN query against available genome sequences in Phytozome, NCBI, TAIR and Congenie databases [18–21]. The search included genomes of unicellular green algae (4 species), nonvascular plants (Bryophyte - 1 species), seedless plants (Lycopodiophyta - 1 species), and seeded plants: Gymnosperms (Gnetophyta - 1 species; Coniferophyta - 1 species; Ginkgophyta - 1 species) and Angiosperms (22 species). Redundant sequences were removed manually. In addition, the putative orthologs in Arabidopsis of each protein containing Agenet/Tudor Domain were identified by TBLASTN in TAIR (Additional file 7).

In total, 31 species were studied, from green algae to angiosperms, as it was summarized in Additional file 1. The analysis revealed that lower plants such as green algae and moss have none or fewer Agenet/Tudor genes compared to those of higher plants. Only one member of the Agenet/Tudor family was found in *Coccomyxa*, four members were found in *Physcomitrella patens*, and above ten members were identified in most of the higher plants. This data suggested that the number of Agenet/Tudor family members expanded in plant genomes with the evolution of plants.

### Phylogenetic Analyses of proteins containing Agenet/Tudor domains in the plant kingdom show key ramifications in higher plants

**Fig. 1:**
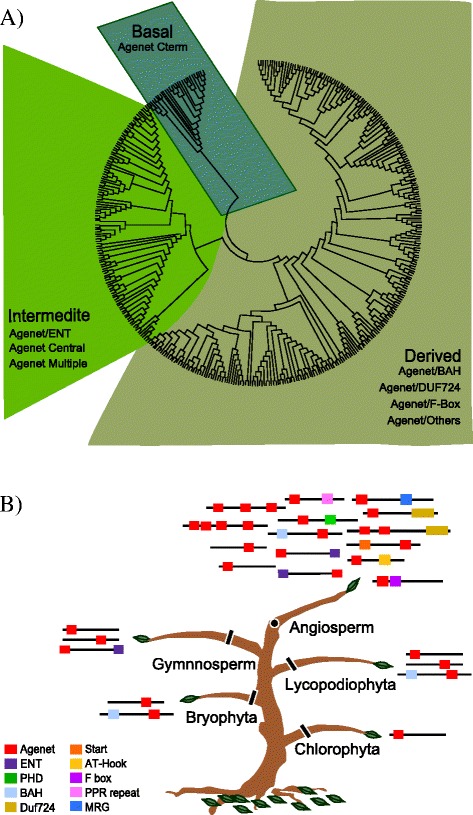
Phylogenetic analysis of the family of Agenet/Tudor proteins in the plant kingdom. **a** Phylogenetic analysis represented as a simplified version of the neighbor joining (NJ) tree, with 416 sequences of proteins from 31 species, from green algae to angiosperms. The tree was divided into three clades: a) the Derived clade, containing 279 sequences from 26 species, that harbor Agenet/Tudor domains combined with BAH, DUF724, F-box and other domains; b) the Intermediate clade, containing 111 sequences from 25 species, that harbor repetitions of Agenet/Tudor domains at N-term or central, combined or not with ENT domain; c) the Ancient (basal) clade, containing 29 sequences from 20 species, harboring one Agenet/Tudor domain in the C-term. **b** Schematic representation of the distribution of members of the Agenet/Tudor Family, the diversity of co-occurring domains and their phylogenetic relationships. There were 442 sequences of 33 species in 24 families from green algae to angiosperms. The squares represent the domains present in the proteins and the colors specify the domains according to the legend. A few rare domains are not represented. The species are listed in Additional file 7

To investigate evolutionary changes of proteins containing Agenet/Tudor domains, phylogenetic analyses using the full-length sequences of 386 domains from 30 species from green algae to angiosperms were conducted. Some bootstrap values for interior branches were low because of the large number of sequences included [39]. A relatively well-supported phylogenetic tree could be constructed after removing all Arabidopsis proteins, possibly due to the large amount of noise these very diverse sequences caused in the program while resolving the analysis (Fig. 1a). The members of the Agenet/Tudor family were grouped in three main clades separated by their conserved domains other than Agenet/Tudor. The three clades were: a) the derived clade, containing 279 sequences from 26 species; b) the intermediate clade, containing 111 sequences from 25 species; c) the ancient (basal) clade, containing 29 sequences from 20 species.

To further investigate the evolutionary relationships observed between Agenet/Tudor members of the three clades, a search for conserved domains was also performed for all sequences using Pfam [22] and SMART [23] with *e*-value cutoff of > *e-5* for domain identification. The number of Agenet/Tudor domains and their position (N-terminal, central or C-terminal) was annotated for each sequence, as well as other domains that may co-occur with Agenet/Tudor domain (Fig. 1b and Additional file 7). The analyses revealed that the first basal Agenet/Tudor domain did not co-exist with other domains in the same protein. Nevertheless, in Bryophyta and Lycopodiaophyta the Agenet/Tudor got combined with BAH domains. In Gymnosperms it co-exists with ENT. Finally, in Angiosperms, the family was enriched with Agenet/Tudor repetitions and the presence of other classes of domains in the same protein structure.

### Plant Agenet/Tudor domains are structurally very similar to the animal Tudor domain

**Fig. 2:**
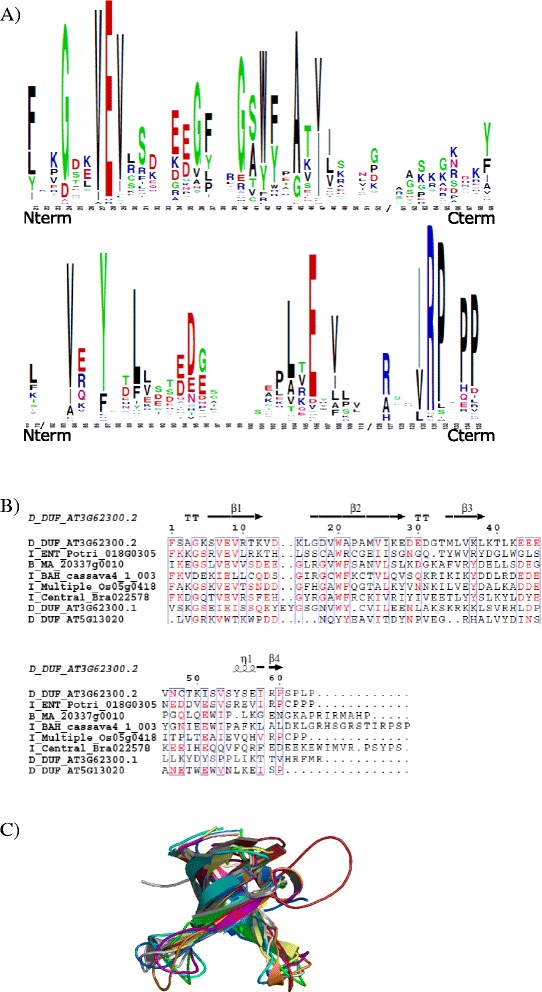
Signature and predicted structure of Agenet/Tudor Domain from Arabidopsis proteins. **a** Alignment of Agenet/Tudor sequences from Arabidopsis showing the canonical conserved residues analyzed by WebLogo. The highly conserved residues are represented as larger letters in the sequence. Although very diverse, some key-positions contain conserved amino acids and possibly maintain the conserved secondary structure observed. **b** Multiple sequence alignment of Agenet/Tudor domain sequences from plant proteins: the alignment was performed using Multalin and the result submitted to ESPript server to plot the secondary structure information of the conserved domains over their primary sequence. On the secondary structure displayed, 3_10_-helices are represented as small squiggles (Ƞ), β-strands are rendered as arrows, and strict β-turns (TT). On the primary sequence alignment, the red characters represent similarity of the amino acid residues in the same group of one column and the blue frame represents the similarity across groups. The sequences used for structural analysis and computer modeling were chosen to represent all clades of plants: Gymnosperm *Picea abies* MA_20337g0010; Angiosperm Monocot *Oryza sativa* Os05g04180; Angiosperm Eudicot *Populus trichocarpa* Potri_018G030500_5, *Brassica rapa* Bra022578, *Manihot esculenta* cassava4_1_003152, *A. thaliana* AT3G62300, AT5G13020. The two sequences of Agenet/Tudor repetitions from AIP1 were used (AT3G62300.1 and AT3G62300.2). **c** Overlapping Agenet/Tudor models generated in the I-TASSER server. The structures are colored in white (B_MA_20337g0010), purple (I_ENT_Potri_018G030500_5), firebrick (I_Central_Bra022578), orange (I_Multiple_Os05g04180), blue (I_BAH_cassava4_1_003152), cyan (D_DUF_AT3G62300.1), yellow (D_DUF_AT5G13020), and green (D_DUF_AT3G62300.2)

Agenet/Tudor domain has been previously classified as a member of the Royal family of domains, and Agenet/Tudor was described as a Tudor-like plant domain [3]. Previously, it has been reported that the Agenet/Tudor domains from Arabidopsis proteins contain an average of 60 amino acids within a few conserved positions and a distant relation based on sequence alignment with Royal family domains [3]. In order to construct a general signature for Agenet/Tudor domains in the plant kingdom, a multiple sequence alignment of the 54 most distinguished Agenet/Tudor sequences found in plants was performed to determine the canonical conserved residues that were analyzed by WebLogo (Web-based sequence logo generating application; Weblogo.berkeley.edu) (Fig. 2a). The Agenet/Tudor domain signature from plants has a few conserved amino acids (at least 16 aa) through the domain sequence within 51 to 101 aa length, and it was very similar to the Logo constructed based only on Arabidopsis*’*s Agenet/Tudor domains and FRMPs from animal. The Agenet/Tudor domain signature revealed that the primary sequences of this domain are very variable among different proteins. In order to investigate the structural homology of the Agenet/Tudor domains from plant proteins, first the characteristic of secondary structure was built by aligning different Agenet/Tudor proteins from different plants using Multalin [29] and ESPript [30]. The secondary structure was characterized by strict β-turns, four beta-sheets and a 3_10_-helices (Fig. 2b – see parameters data in Additional file 8), (similar to the information about secondary structure in reference 3). Next, the structural homology among the same Agenet/Tudor sequences was evaluated using I-TASSER [27]. All Agenet/Tudor models produced had shown significant parameters of C-score and TM-score (See Additional file 9) and the characteristic structure of tudor-like Beta-barrel folding was suggested to be conserved in the plant Agenet/Tudor models proposed in this study (Fig. 2c). The individual structures are represented in Additional file 2. All together, the secondary structure models predicted in this work showed that the plant Agenet/Tudor domains might be, in general, very similar between themselves, indicating that they may belong to a consistent family of protein domains despite their low identity in amino acid sequences.

### The Agenet/Tudor family in Arabidopsis has four different classes based on domain organization

**Fig. 3:**
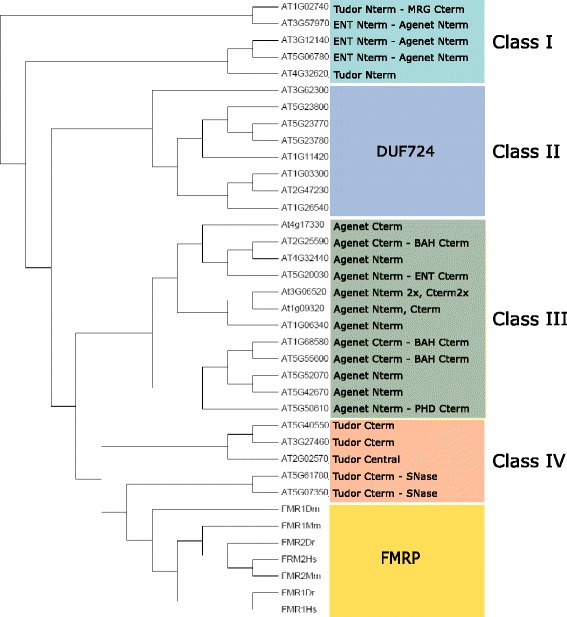
Phylogenetic classification of the Agenet/Tudor family in *Arabidopsis.* The phylogenetic tree (NJ) was constructed by MEGA6 using the members found in Arabidopsis and the proteins FMR1 and FMR2 of *D. melanogaster, M. musculus, D. rerio* and *H. sapiens* as roots (Additional file 7)

In order to better understand the phylogeny of Agenet/Tudor containing proteins from Arabidopsis, the 30 sequences from the family members were used to construct a tree in Mega 6.0 program [24]. The FMRPs from human, mouse, fly and zebra fish sequences from NCBI [19] were also used. A paraphyletic tree focusing on functional characterization was constructed and allowed the visualization of distinct branches from which the proteins were classified based on the organization of their domains. The Agenet/Tudor class I has N terminal Agenet/Tudor domains and some members also harbor the ENT domain. Class II proteins co-occur with DUF724 domain in the C-terminus. Class III has more diverse members with Agenet/Tudor domains in N and/or C terminal positions, multiple Agenet/Tudors repetitions or co-exist with BAH or PHD. Class IV proteins are the most similar to the animal FMRPs (Fig. 3).

**Fig. 4:**
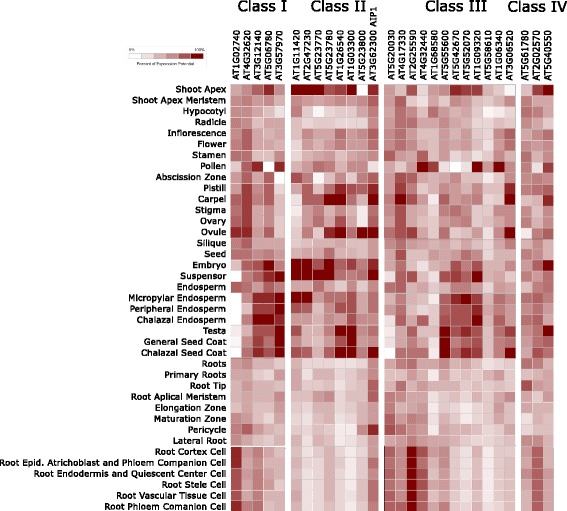
Expression profile of members assigned in each Class of Agenet/Tudor family in Arabidopsis. The expression pattern is showed in different plant tissues and organs as a heat map representation of the average values among the expression values published in many microarray experiments available in Genevestigator (https://genevestigator.com) [40]. The genes AT5G07350 and AT3G27460 from Class IV are out of analysis since there are no probes in the available microarray data

To investigate possible developmental processes in which the distinct classes of Agenet/Tudor genes in Arabidopsis could participate, their expression pattern was searched *in silico* through Genevestigator database [40]. In general, members of the Agenet/Tudor family were highly expressed in reproductive tissues as seed and embryo (Fig. 4). The different Agenet/Tudor family classes showed some particularities in the expression profiles of their members (Fig. 4). Class I genes were highly expressed in seed and embryo tissues. Class II were likewise found in seed and embryo, but were also highly expressed in shoot apex and flower female tissues (as carpel and ovules). The expression of Class III members was distributed among different plant organs and tissues, with some genes being more expressed in pollen and seed. From the five members of Class IV, two genes were not represented in microarray data experiments, invalidating analysis of patterns. The temporal expression of Agenet/Tudor domain proteins during development was also analyzed *in silico* through Genevestigator database (Additional file 3). Class I members showed moderate levels of expression with almost no variation during development, and increased mRNA levels were observed in late maturation of seeds and senescence of leaves. Class II members also exhibited moderate expression levels, peaking during bolting phase and embryo maturation phase. Expression profile of Class III members was again very diverse.

Interestingly, the Arabidopsis Agenet/Tudor genes were highly expressed in reproductive tissues and evolutionary analysis showed a dramatic increase of members and domains diversity of Agenet/Tudor family in the flowering plants (Fig. 1b). All together, the data suggests a possible role of Agenet/Tudor domain proteins during flower development and embryo formation.

### Identification of AIP1 as an Agenet/Tudor/DUF724 domain protein that interacts with ABAP1

**Fig. 5:**
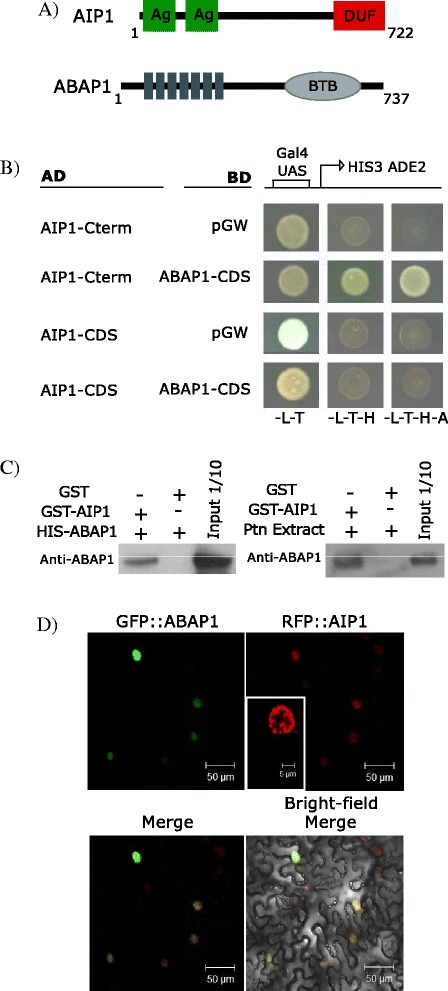
Characterization of AIP1 protein interactions and subcellular localization. **a** Schematic representation of AIP1 and ABAP1 proteins. AIP1 harbors two repetitions of Agenet/Tudor (Ag) domains in its N-Terminal and a DUF724 in the C-terminal (DUF); ABAP1 harbors eight Beta-catenin-type Armadillo (ARM) at its N-terminal and one BTB/ POZ (BTB) domain in the C-terminal. **b** Yeast two hybrid assays with the C-terminal region of AIP1 (aa 540-723) or the complete AIP1 CDS fused with GAL4 DAD (AIP1-C-Term AD and AIP1-CDS AD, respectively) against full-length ABAP1 fused with GAL4 DBD. GAL4 DBD empty vector was used as negative control. Details of the constructs can be found in Additional file 13. Yeast transformation was selected in -L-T (SD medium lacking Leucine and Tryptophan), and protein interactions were selected in -L-T-H or -L-T-H-A (SD medium lacking Leucine, Tryptophan and Histidine, or Histidine and Adenine. **c** Left: GST pulldown of bacterially expressed recombinant GST-AIP1and HIS-ABAP1. Right: Semi-in vivo pulldown assay of bacterially expressed recombinant GST-AIP1 and protein lysates of Arabidopsis 10-day-old plants. ABAP1 interacting proteins were assayed with antibodies anti-ABAP1 in immunoblots. **d** Subcellular localization of GFP::ABAP1 and RFP::AIP1 in abaxial epidermis of *N. benthamiana* 14-day-old leaves by confocal microscopy. RFP::AIP1 inset showing the speckle-pattern in nucleus

To search for proteins that could participate with ABAP1 in the control of DNA replication and transcription, a yeast two-hybrid screen was performed with an Arabidopsis cDNA library using ABAP1 as bait [17]. Members of transcription factors families were identified, such as TCP24, which acts together with ABAP1 regulating cell division in leaves [17]. Among the ABAP1-interacting proteins (AIPs) identified, there was AIP1 (At3G62300), an unknown protein predicted with 722 amino acids and approximately 80,9 kDa. It harbors two repeats of Agenet/Tudor domain in its N-terminal region (amino acids 13–84, and 161–224) as well as a DUF724 domain in its C-terminus (amino acids 540–722) (Fig. 5a). The Agenet/Tudors domain is 63 and 71 amino acids long and the DUF724 domain is 182 amino acids long. Previous studies on DUF724 gene family of Arabidopsis described Agenet/Tudor as an RNA-binding domain based on its similarity to animal Tudor domain from FMRP and named AIP1 as DUF7 [4]. AIP1 belongs to Class II of Agenet/Tudor family in Arabidopsis, together with others Agenet/Tudor/DUF724 proteins (Fig. 3).

The interaction between AIP1-ABAP1 in yeast two-hybrid assays was mapped within the C-terminus region of AIP1 (amino acids 532–723) that contains the DUF724 domain and the N terminus region of ABAP1 (amino acids 1–350) that contains the Beta-catenin-type Armadillo repeats (ARM repeats) (Fig. 5b and Additional file 4). Surprisingly, the full-length AIP1 did not interact with ABAP1 in the yeast two-hybrid assay (Fig. 5b). Nevertheless, the association between ABAP1 and the full length AIP1 was confirmed in GST pulldown experiments with HIS::ABAP1 and GST::AIP1 (Fig. 5c), and it was further confirmed in semi-in vivo pulldown assays with GST::AIP1 and protein extracts of 10 day-old Arabidopsis plants (Fig. 5c).

AIP1 does not exhibit any clear DNA-binding signature and no signal peptide prediction by iPSORT search. Co-transfection experiments with RFP::AIP1 and GFP::ABAP1 in *Nicotiana benthamiana* leaf abaxial epidermis confirmed the nuclear localization of AIP1 [4], and showed co-localization with ABAP1 (Fig. 5d). Confocal microscopy images indicated that AIP1 was exclusively located in the nucleus, and enriched in nuclear domains (Fig. 5d). Remarkably, ABAP1 was also reported to be exclusively located in the nucleus, homogeneously distributed or enriched in nuclear domains in a speckle pattern [17].

All together, the data suggests that AIP1 could participate with ABAP1 in regulatory complexes. An important question to be addressed is whether AIP1 has a role on chromatin remodeling during ABAP1’s regulation of DNA replication and/or gene expression.

### AIP1 Agenet/Tudor domain is most similar in structure to a Tudor domain that functions as histone modification reader

**Fig. 6:**
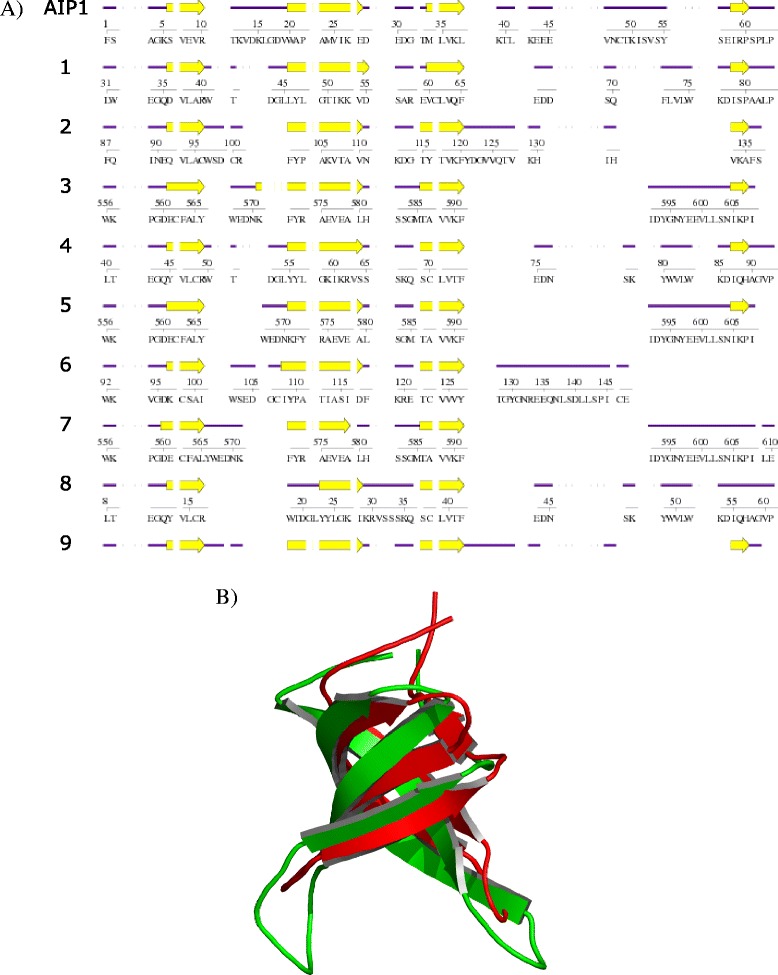
Predicted Secondary Structure Matching of Agenet/Tudor domain from AIP1 with different Tudor domains. **a** Alignment of secondary structures performed by ProFunc using Agenet/Tudor sequence from AIP1 as query. The proteins shown are: 1. PHF1 Tudor in complex with H3K36me3 by X-Ray Diffraction; 2. Crystal structure of Tudor domain 2 of human PHF20 by X-Ray Diffraction; 3. Tudor domain of human TDRD3 (Tudor domain- protein 3) by X-Ray Diffraction; 4. Solution NMR (Nuclear Magnetic Resonance) for human PHF19 linking H3K36me; 5. Tudor domain of human TDRD3 by X-Ray Diffraction; 6. Human Tudor domain of SMN1 in complex with aa organic molecule by X-Ray Diffraction; 7. Human TDRD3 complex with asymmetric dimethylarginine mark in histone by Solution NMR; 8. Solution NMR structure of the human Tudor domain of PHF19, isoform b; 9. The second Tudor domain of human PHF20 by X-Ray Diffraction. **b** Modeled structure of the Agenet/Tudor domain of AIP1 (Green) superimposed to the Tudor domain of PHF1 (Hit 1. PDB id: 4HCZ) (Red)

To get insights into the function of the Agenet/Tudor domain from AIP1, we first addressed how close AIP1 domain is to the animal Tudor domains, by performing computer structural modeling. For ProFunc analysis [32], the second repetition of Agenet/Tudor domain in the N terminus from AIP1 was used as query. The analysis showed that all structures from the Protein Data Base (PDB) with higher scores were found in animal Tudor domains (Fig. 6a). Since all Agenet/Tudor modeled domains may have a conserved Tudor-like Beta-barrel folding, the Agenet/Tudor and Tudor domains might have similar folding and structure. The root-mean-square deviation (RMSD) between the C-alpha atoms and the statistic relevant z-score of the compared structures (see Additional file 8) insure the significance of the hits found. The best outcome (in red cartoon representation) was superposed to the AIP1 N terminus Agenet/Tudor domain (in green cartoon representation) and a directly similarity can be observed (Fig. 6b). The most similar Agenet/Tudor domain structure (mentioned as number 1 in Fig. 6a) is present in the PHD Finger protein 1 (PHF1), a *polycomb group* (*PcG*) gene that is a histone modification reader known to specifically bind to histone H3K36me3 and to recruit the Polycomb Repressive Complex 2 (PRC2) in humans [41]. *PcGs* are known to silence expression mostly thought regulation of chromatin structure, in part through post-translational modification of histones [42].

### AIP1 is highly expressed in reproductive tissues

**Fig. 7:**
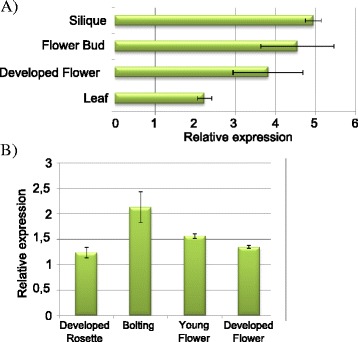
AIP1 expression in different Arabidopsis tissues and organs. Relative mRNA levels of AIP1 were determined by qRT-PCR in **a** different organs from 30 day-old plants; except leaves which have been harvested from 14 day-old plants; **b** entire plants in different stages of development. Values were normalized with AtUBI14 as reference gene. Data shown represent mean values obtained from independent amplification reactions (*n* = 3) and biological replicates (*n* = 2). Each biological replicate was performed with material collected from a pool of at least six plants. Bars indicate mean ± standard error of biological replicates

To identify possible functions for AIP1 during plant development, its gene expression profile was analyzed *in silico* in open access microarray databases and by qRT-PCR assays (Fig. 7). High *AIP1* expression was observed in various reproductive tissues, such as carpel and in seed tissues such as chalazal seed coat of globular and heart shape embryos [43] and in the suspensor, where its expression was about 4,5 times higher than in others tissues [44] (Fig. 4). qRT-PCR confirmed a high expression of *AIP1* in siliques and flower buds in comparison to developed flowers and leafs (Fig. 7a). The shoot apex meristem also showed high *AIP1* mRNA levels [45] (Fig. 4). A peak of *AIP1* expression was observed during bolting (Fig. 7b), the timing of development that marks the transition from vegetative to reproductive phase, and a second peak of expression was observed during seed development. All together, the data revealed a peak of expression of *AIP1* during transition of vegetative to reproductive phase of development, with the main expression occurring in early flower development, especially in female organs, suggesting that AIP1 might have, amongst other functions, a role during plant reproductive phase.

### AIP1 interacts with ARIA, an ABAP1 homologue in Arabidopsis, and with LHP1, a chromatin remodeling protein

**Fig. 8:**
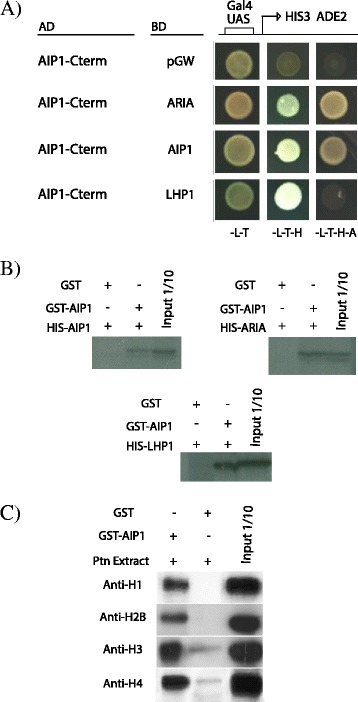
AIP1 protein interactions. **a** Yeast two hybrid assay with the C-terminal of AIP1 (aa 540-723) fused with GAL4 DAD (AIP1-C-Term AD;) against ARIA, AIP1 and LHP1 full-length CDSs fused with GAL4 DBD. GAL4 DBD empty vector was used as negative control. Details of the constructs can be found in Additional file 13. Yeast transformation was selected in -L-T (SD medium lacking Leucine and Tryptophan), and protein interactions were selected in -L-T-H or -L-T-H-A (SD medium lacking Leucine, Tryptophan and Histidine, or Histidine and Adenine. **b** GST pulldown of bacterially expressed recombinant GST-AIP1 with HIS-AIP1, or HIS-ARIA, or HIS-LHP1. HIS-tag interacting proteins were assayed with antibodies anti-HIS in immunoblots. **c** Semi-in vivo pulldown assay of bacterially expressed recombinant GST-AIP1 and protein lysates of Arabidopsis 10-day-old plants. Histone interacting proteins were assayed with Anti-H1, Anti H2B, Anti-H3 and Anti-H4 antibodies in immunoblots

To obtain further insights into AIP1 function, other interacting proteins and complexes in which AIP1 takes part were searched using yeast two-hybrid assays. AIP1 interaction with other proteins related to the ABAP1 network, pre-RC members and chromatin remodeling proteins was tested using the AIP1 C terminus region harboring the DUF724 domain, since the full-length AIP1 was not able to establish protein-protein interactions in the yeast two-hybrid assay. The complete list of pair interactions tested by yeast-two hybrid is listed in Additional file 10. Differing from ABAP1, AIP1 did not show positive interactions with proteins that are part of the pre-RC. Interestingly, AIP1 C-term was able to form homodimer with the AIP1 full-length protein in the yeast-two hybrid assay (Fig. 8a) and the association of the full-length AIP1 proteins was observed in GST pulldown assays with GST::AIP1 and HIS::AIP1 (Fig. 8b). AIP1 also interacted with ARIA - the ABAP1 homolog (Fig. 8a, Additional file 4) and a weak interaction between the DUF724 domain of AIP1 and the LHP1 full-length protein was identified by yeast-two hybrid (Fig. 8a). These interactions were further confirmed by GST pulldown assays with CDS sequences in fusions: GST::AIP1 and HIS::ARIA or HIS::LHP1 (Fig. 7b). The association with LHP1 supports a possible role of AIP1 protein on chromatin remodeling, since it is suggested that LHP1 is a regulator of gene expression by controlling chromatin packaging depending on the status of methylation of its histones [46].

### AIP1 interacts with non-modified Histones

Next, a tandem affinity purification (TAP) assay was performed using AIP1 as bait willing to identify protein complexes formed in vivo (see Methods). However, TAP was hampered by the difficulty to well express full length AIP1 fused to the affinity tag in cell cultures. Nevertheless, the output from the TAP purification assays was a small portion of a conserved sequence of the Histone superfamily (Additional file 11). Since AIP1 is member of the Royal domain family that interacts with the chromatin remodeler LHP1 and with the DNA replication and transcription regulator ABAP1, it is reasonable to expect that AIP1 could bind to histones, whether they are modified by methylation or acetylation, or none. To further investigate a possible association between AIP1 and histones, a semi-in vivo pulldown assay was performed using full-length GST::AIP1 and protein extracts of Arabidopsis 10 day-old plantlets, and the AIP1 interacting proteins were assayed with antibodies against specific histones (Fig. 8c). The results showed that AIP1 could bind to the non-modified histones H1, H2B, H3 and H4. However, it couldn’t bind to the two forms of acetylated histones, H3K9ac and H3K14a (Additional file 5), that are recognized in maize by the Agenet/Tudor/ENT domain protein RIF1 [13], suggesting that the two Agenet/Tudor members might have evolved different roles on plant development.

### AIP1 down regulation delays flower maturation

**Fig. 9:**
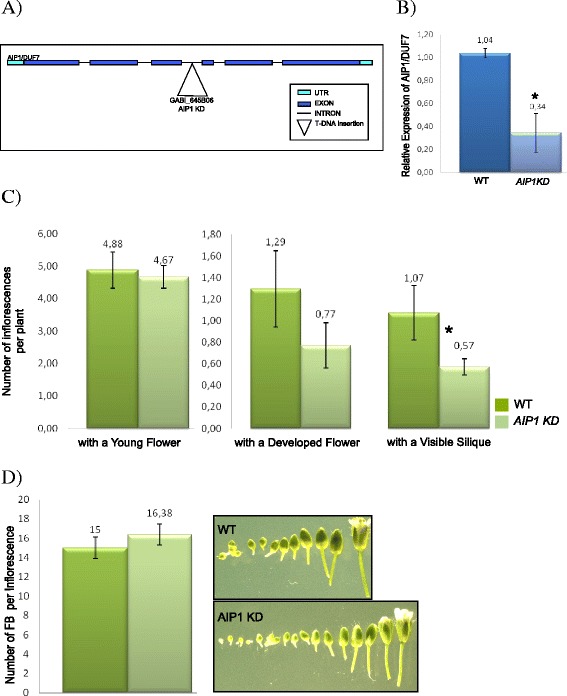
Molecular and phenotypic analyses of AIP1^KD^ lines. **a** Schematic representation of AIP1^KD^ line (GABI_645B06) indicating the T-DNA insertion in the third intron. **b** Relative mRNA levels of *AIP1* in 21 day-old WT and *AIP1* ^*KD*^ homozygote plants were determined by qRT-PCR. Data were normalized with *AtUBI14* as reference gene. Data shown represent mean values obtained from independent amplification reactions (*n* = 3) and biological replicates (*n* = 2). Each biological replicate was performed with material collected from a pool of at least six plants. Bars indicate mean ± standard error of biological replicates. A statistical analysis was performed by *t*-test (*p*-value <0.05). Asterisks (*) indicate significant changes between samples. **c** Comparative analyses of the number of reproductive structures in 30 day-old *AIP1* ^*KD*^ and WT plants. The graphs show the number of inflorescences containing young flowers per plant (left), inflorescences containing developed flowers per plant (middle), and inflorescences containing visible siliques per plant (right). Data has been quantified in ten plants. Values shown are means derived from two independent experiments. Bars indicate mean ± standard error of biological replicates. A statistical analysis was performed by *t*-test (*p*-value <0.05). Asterisks (*) indicate significant changes between control (wild-type) and samples. **d** (Left panel) Comparative analyses of developmental timing of flowers in 30 day-old *AIP1* ^*KD*^ and WT plants. Values shown are means derived from two independent experiments. Bars indicate mean ± standard error of biological replicates. A statistical analysis was performed by *t*-test (*p*-value was 0.06). (Right panel) Photographs of dissected representative inflorescences of WT and AIP1^KD^ plants

To access the function of AIP1 during Arabidopsis development, plants with reduced or silenced expression levels of *AIP1* were searched in the collections of T-DNA insertion mutants. No SALK T-DNA insertions were found in *AIP1*. A homozygote GABI line mutant (GABI_465B06) with the T-DNA inserted in the third intron was characterized (Fig. 9a). *AIP1* expression levels were around three fold decreased in GABI_465B06 homozygote plants (here denoted as AIP1^KD^) (Fig. 9b).

The phenotype of *AIP1*^*KD*^ plants was analyzed all over development, and it was compared with wild type control lines. During vegetative development, *AIP1*^*KD*^ plants with lower levels of *AIP1* developed normally and no significant difference was observed in leaf area, number of cells and ploidy, as well as in root growth (Additional file 6). During reproductive phase, a slight difference in developmental timing of reproductive organs could be seen in the *AIP1*^*KD*^ mutant, as 30 day-old plants had developed a reduced number of inflorescences with mature flowers and siliques per plant (Fig. 9c). However, these differences disappeared during plant senescence (data not shown), if plants are kept in watering. In an attempt to measure the timing of flower development in 30 day-old *AIP1*^*KD*^ mutants, the number of visible flower buds per inflorescences containing one open-flower in stage 6-12, according to [47], was counted under binocle (Fig. 9d). Although there was a tendency for a delay in flower maturation in the *AIP1*^*KD*^ mutant, it is not statistically different.

Although plants full silenced for *AIP1* could not be obtained, the phenotype of plants with reduced levels of *AIP1* suggested a role of this protein in flowering and reproduction, and it is consistent with high expression levels of *AIP1* in this phase of plant development. In addition, AIP1 possible orthologs were found only in Angiosperm species (highlighted in yellow in Additional file 7).

### Down regulation of AIP1 increases expression levels of ABAP1 and LHP1 target genes

In order to investigate if AIP1 could act together with ABAP1 and LHP1 during flower development, mRNA levels of ABAP1 and LHP1 target genes were analyzed in flower buds of plants with reduced levels of AIP1 (AIP1^KD^) compared to WT control plants, by qPCR.

**Fig. 10:**
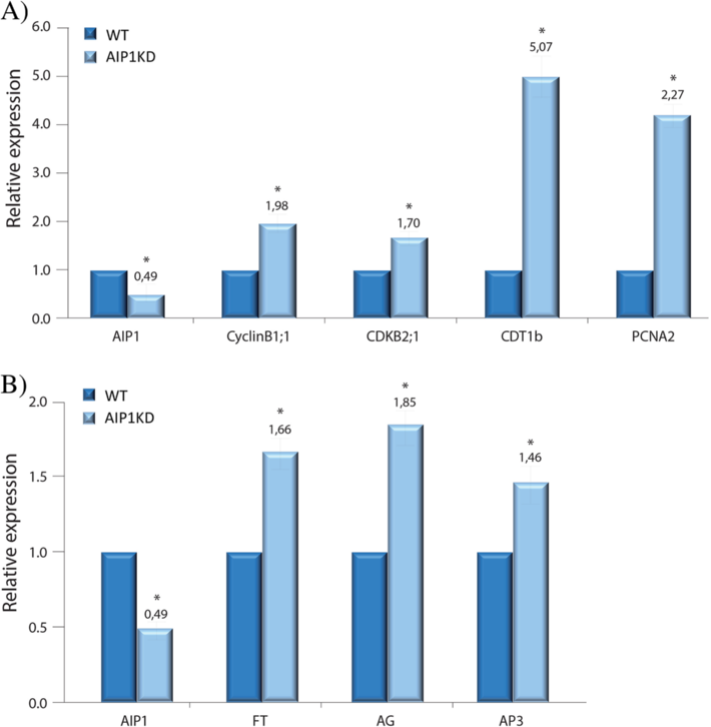
Gene expression in *AIP1* ^*KD*^ homozygote plants. Relative mRNA levels in flower buds of WT and *AIP1* ^*KD*^ homozygote plants were determined by qRT-PCR of (**a**) *AIP1, CDT1b, CyclinB1;1, CDKB2;1* and *PCNA;* and (**b**) *AIP1, FT, AG* and *AP3*. Data were normalized with *AtUBI10* and *GAPDH* as reference genes. Data shown represent mean values obtained from independent amplification reactions (*n* = 3) and biological replicates (*n* = 3). Each biological replicate was performed with material collected from a pool of at least six plants. Bars indicate mean ± standard error of biological replicates. A statistical analysis was performed by *t*-test (*p*-value <0.05). Asterisks (*) indicate significant changes between samples

To verify a possible role of AIP1 in DNA replication, expression of Cdt1b, one component of the pre-RC that is a target of ABAP1 transcription repression (Masuda et. al., [17]) was investigated. Besides being transcriptionally regulated, Cdt1b is a DNA replication marker, as well as *Proliferating Cell Nuclear Antigen 2* (PCNA2), another S phase marker gene that is responsible to restore replication fork progression when DNA is damaged [48]. Also, the expression of G2-M transition and cell division markers, as CyclinB1;1 and CDKB2;1 was analyzed [49]. As shown in Fig. 10, AIP1 expression levels were reduced by approximately 50 % in AIP1^KD^ flower buds compared to control plants. On the other hand, Cdt1b and PCNA2 mRNA levels were highly increased in AIP1^KD^ flower buds, suggesting that AIP1 might operate with ABAP1 negatively regulating DNA replication. An increase in CyclinB1;1 and CDKB2;1 mRNA levels were also observed in AIP1^KD^ flower buds (Fig. 10a), indicating that the DNA replication stimulus is possibly followed by an increase in cell proliferation rates and/or a delay in cell differentiation in AIP1^KD^ mutants.

To test a role of AIP1 together with LHP1 in flower development, the expression of genes epigenetically repressed by LHP1, known as floral development target genes such as Flower Locus T (FT), Agamous (AG) and APETALLA 3 (AP3) [50], was analyzed. As shown in Fig. 10b, mRNA levels of FT, AG and AP3 increased in AIP1^KD^ flower buds, compared to control. All together, the data showed that AIP1 interacts with ABAP1 and LHP1 and possibly participates in the repression of expression of some of their target genes during flower development.

## Discussion

The dynamics of chromatin modification and accessibility is likely to exert a key role in regulating DNA replication and transcription, critical cellular processes pivotal for modulating plant growth and development. However, how these processes are coordinated and integrated with developmental signals has not yet been fully clarified. In this work we investigated a family of chromatin remodeling proteins in plants, containing the Agenet/Tudor domain, described as histone modification “readers”. This class of proteins is still poorly characterized in the plant kingdom. We identified and characterized a novel member, named AIP1 and previously called DUF7 [6], which can further reveal insights into mechanisms connecting DNA replication, gene transcription and chromatin remodeling.

### Plant proteins with Agenet/Tudor domain could have acquired roles in developmental processes occurring during plant reproductive phase

In this study, 416 proteins containing the Agenet/Tudor domain in plants, including at least 380 previously undefined ones, were described. Our analysis revealed just a single protein containing the Agenet/Tudor domain in one out of four Green Algae species analyzed, suggesting this family got emerge since then. Phylogenetic analyses using the 386 members of Agenet/Tudor family from 30 plant species supported that the Agenet/Tudor family has a monophyletic origin. Previous studies on the DUF724 protein family, comprising some Agenet/Tudor domain proteins from *A. thaliana*, rice, poplar and *Vitis vinifera,* also supported the common ancestral origin for this family [4].

Agenet/Tudor domain has been previously classified as a member of the Royal family of domains, and Agenet/Tudor was described as a Tudor-like plant domain [3]. During many years Tudor was described as a RNA binding domain [51, 52], with some involvement in interaction with modified histone tails, and in DNA repair and cell cycle, but without a clear understanding of its function [53–55]. It was from the observations on the plant Agenet/Tudor domain [4, 13, 14, 56, 57] that it was recently possible to fully comprehend the Human Tudor structure and function [5, 7–10]. It was elucidated that the Tudor domain is not the responsible for interacting with nucleic acids, but a neighbor domain called KH, present in some of the Tudor proteins [5]. Although Agenet/Tudor domains have been described as an RNA binding domain [4], this activity has not been experimentally demonstrated. Furthermore, the KH domain have not been found in plant Agenet/Tudor proteins so far; however the prediction of this domain depends on the use of more sophisticated protein structure tools and might thus been overlooked. One exception is the AtCoilin protein that has three sites that bind to RNA, but none are located in the Agenet/Tudor domain region [14]. The prediction of KH domain depends on the use of more sophisticated protein structure tools, therefore it is reasonable to expect that other plant Agenet/Tudor proteins also contain the KH domain, and are able to bind histones and RNAs.

We found evidences to suggest that plant proteins containing the Agenet/Tudor domain alone or coexisting with the BAH domain represent the oldest members in an evolutionary scale. Green algae contain a single protein with only one Agenet/Tudor domain, whereas in Bryophyte and Lycopodiophyte the Agenet/Tudor domain co-occurs with the BAH domain. Later in evolution, in Gymnosperms, the ENT domain can co-occur with the Agenet/Tudor domain. Finally, a “boom” of diversity of forms and sequences of Agenet/Tudor proteins has occurred in Angiosperm species that might be correlated with a functional diversification of Agenet/Tudor proteins during plant reproduction and processes of flower development. Corroborating with a role of members of this family in plant reproductive phase, the expression profile of Agenet/Tudor family in Arabidopsis showed main expression in reproductive tissues and embryo.

Some of the animal Agenet/Tudor domain proteins have been implicated in having a role during gametogenesis. The Tudor-SN (TSN) protein of Drosophila is involved in oogenesis [51] and five from eleven copies of its Tudor domains are sufficient for the germ cell formation [58]. Although the mechanism of action of TSN is still unclear, it is part of the RISC complex that regulates RNA silencing [52]. FMRP’s Tudor domains can bind to chromatin through H3K79me to respond to DSB by regulating the deposition of a variant of histone, yH2A.X, and mutations in this gene lead to defects in gametogenesis [6]. In Arabidopsis, it was suggested that the ENT/Agenet/Tudor proteins denominated AtEMLs, together with EDM2, may link race-specific pathogen recognition to general defense mechanisms through chromatin remodeling processes [12]. Also, EDM2 positively affects floral transition by suppressing *FLC* expression [12], and *EML1* and *EML2* mutants have and early-flowering phenotype [12]. Another well-described regulator of *FLC* is LHP1, a chromo domain containing protein involved in chromatin regulation of flower timing in the apical meristem of Arabidopsis. LHP1 recognizes and binds to H3K27Me3, epigenetically regulating the levels of *FLC* before and after the vernalization period [59], as well as other MADS box genes [60]. LHP1 homologs were found only in Angiosperms, suggesting that LHP1 role in flower timing and development might be spread in flowering plant species [46].

We also found that AIP1’s homologues are exclusively found in the Angiosperm clade, supporting the idea of a function in flower timing and/or development. Moreover, AIP1 interacts with LHP1 and both are expressed in the shoot apical meristem and reproductive tissues, suggesting they may share a role in these organs. Moreover, our studies showed that mutant plants with decreased expression of *AIP1* exhibited higher expression levels of flower development genes epigenetically regulated by LHP1, and these plants also showed a small delay in the timing of flower development, forming inflorescences with at least one flower bud more, compared to wild type. The phenotype was subtle and occurred during just a brief window of time, possibly because around 40 % of *AIP1* mRNA levels were still expressed in these plants and/or there is redundancy in the regulation of the process. Mutants in the gene *CORYMBOSA2* (*CRM*2), encoding a methyltransferase of miRNAs and siRNAs, have a phenotype with little effect on the timing of floral induction, but showing notably a delay in the development of flowers [61]. As a result, *crm2* mutants have an increased number of flower buds in the inflorescences [61], similarly as the phenotype observed in *AIP1*^*KD*^ mutant. Even though the mechanism of action is still not known, it seems reasonable to hypothesize that an epigenetic regulation of gene expression is affecting flower development process in both mutants.

### AIP1 is an Agenet/Tudor protein in plants that might connect cell cycle and chromatin remodeling processes

The involvement of proteins containing Agenet/Tudor domain in chromatin remodeling and cell cycle regulation is still not well understood. In plants, members of this protein family were not yet reported as directly involved in chromatin dynamics during cell cycle. This work characterized in more detail the Agenet/Tudor protein AIP1, that binds to histones and to ABAP1, a regulator of DNA transcription and of licensing DNA to replicate in Arabidopsis [17]. The full-length GST::AIP1 was able to pull down both ABAP1 and histones in a semi-in vivo GST pulldown assays, suggesting that AIP1, ABAP1 and histones might be found in the same complex. qRT-PCR data showed that expression of Cdt1, a preRC gene repressed by ABAP1, is increased in AIP1^KD^; as well as the expression of other cell division markers. These findings support that AIP1 could play a role in cell cycle and/or gene expression regulation together with ABAP1, controlling cellular events during G1 to S phase transition in Arabidopsis. An important issue is to unravel the mechanisms by which Agenet/Tudor integrates these cellular processes. Possibly, it might involve the interaction with histones and chromatin remodeling proteins.

Agenet/Tudor proteins in animals have been described as readers of various histone modifications. The Tudor domain FMRP has been implicated in participating in DNA repair by specifically binding to H3K79me [6]. 53BP1 can control of S phase duration by interacting with the RB protein methylated at K810, maintaining its hypomethylated status [7], as well as it can bind to H4K20me2, a DSB mark [7]. The tandem Tudor domain protein Spindlin1 from humans recognizes H3K4 methylation [15], and can bind to mitotic spindle and respond to DSB [16].

The primary structure of Agenet/Tudor domain can be very variable in plants. It contains approximately 50 to 100 aa length with few (at least 16) conserved amino acids in the primary structure, as shown in the WebLogo signature. However, the structural modeling suggested that the plant Agenet/Tudor domains might be similar between themselves, indicating that they belong to a consistent family of protein domains. Moreover, all plant Agenet/Tudor and animal Tudor domains modeled in this work presented favorable results to a conserved Tudor-like Beta-barrel folding, similar to the previous description for animal Tudor folding [28], suggesting that these domains might have evolved separately but converged to a similar structure and its associated folding. Although plant Agenet/Tudor domain proteins have been implicated in chromatin remodeling processes [6–8], a direct binding to histones has not been reported. In this work, a strong interaction of AIP1 with non-modified histones H1, H2B, H3 and H4 was observed. However, AIP1 did not associate with two forms of acetylated histones tested in semi-in vivo pulldown assay, the H3K9ac and H3K14ac (Additional file 5), which are possibly recognized by the Agenet/Tudor/ENT RIF1 protein from maize [13]. Possibly, AIP1 could recognize different histone modifications, having evolved a different role on plant development. Moreover, we found that AIP1 also interacted with LHP1, a chromo domain protein that recognizes and binds to H3K27Me3 [60]. Moreover, the predicted structure of the AIP1 Agenet/Tudor domain is most similar to the one present in PHF1, known to specifically bind to histone H3K36me3 [41]. Although in this work we identified AIP1 interaction with non-modified histones, it could recognize histone modifications different than the ones tested, and further protein structural and interaction works are needed to address AIP1 mechanism of action in chromatin remodeling.

Recently, it has been reported that Arabidopsis SAWADEE Homeodomain Homolog 1 (SHH1) harbors a domain very similar in structure with Agenet/Tudor, the SAWADEE domain [57]. It shows preferential binding to di- or trimethylated H3K9 but it can also bind non-modified H3K4 [57]. It has been proposed that SHH1 Agenet/Tudor domains may allow the transcription, signalized by H3K4, even though a silence mark (H3K9me2/3) is present [57].

The Tudor protein UHFR1 has an allosteric regulation where in a ground state the C-terminal polybasic region of the protein is folded back onto the Tudor domain repetitions, while the PHD domain binds to unmodified H3. In an active state, a cofactor (phosphatidylinositol phosphate - PI5P) is linked to the polybasic region, which stabilizes the orientation of the Tudor domains, giving access to bind to modified histones (H3K9me3) [8]. AIP1 could bind only non-modified histones or, most probably, the internal folding of the protein and/or the binding to an external molecule may regulate different active states where AIP1 can bind histones in a selective way. A regulation of AIP1 protein interactions by internal folding states is supported by the results observed in yeast two-hybrid assays. Although in vitro and semi-in vivo GST pulldown assays showed interaction between full length AIP1 and ABAP1, ARIA, LHP1 and itself, only homodimers of AIP1 could be observed in yeast two-hybrid assays. Possibly, the native structure of AIP1 needs a structural modification to allow DUF724 to bind to these proteins. We could speculate that this modification might involve the association of the N terminus region of AIP1 with histones, in order to allow DUF724 to bind to the other proteins.

Despite the importance of *Polycomb* genes in animal and plant development, their mechanism of action in plants are still poorly understood [62]. In this work we showed that AIP1 can bind LHP1, a major protein for POLYCOMB REPRESSOR COMPLEX1-like (PRC1-like) functions in plants [63]. Also, the Agenet/Tudor domain of AIP1 has a predicted structure very close to the Tudor domains from PHF1 proteins from mammals that bind to methylated histones necessarily to recruit PRC2 to chromatin [31]. Furthermore, LHP1 was already reported to bind to a member of PRC2, promoting its recruitment to chromatin regions that carry H3K27me3 [64]. The LHP1 binding to H3K27me3 is required for its function and for repression of several PcG protein targets such as FT, AG and AP3 [63], and this work showed that these three genes are up regulated in AIP1^KD^ plants. It has been suggested that interaction between plant PRC2-like and PRC1-like complexes in plants contributes to the inheritance of H3K27me3 during DNA replication and to the maintenance of H3K27me3 levels during interphase [64]. Therefore, we can speculate that, by associating with LHP1 and ABAP1, AIP1 could participate in protein complexes that act via histone modification and chromatin remodeling to regulate gene expression during flower development. Finally, the delay in flower maturation observed in AIP1^KD^ plants could be caused by up regulation of the expression of DNA replication, cell division and flower development genes, leading to unbalanced cell division and cell differentiation rates in the developing flowers.

## Conclusions

The phylogenetic and expression analysis of plant proteins containing Agenet/Tudor domain suggest that they might have acquired roles during plant reproduction. We propose that AIP1 is a novel member of the Agenet/Tudor family that might has a role in interconnecting the processes of DNA replication and/or DNA transcription with the dynamics of chromatin accessibility, to regulate flower development in Arabidopsis*.* This hypothesis is supported by the finding that AIP1 interacts with histones, ABAP1, ARIA and LHP1; moreover AIP1, ABAP1 and histones seems to be found in vivo in the same complex. Also, the predicted structure of the Agenet/Tudor domain of AIP1 is most similar to those of PHF1 proteins from mammals that recruit PRC2 to chromatin; and LHP1 binds to a member of PRC2 in plants, promoting its recruitment to chromatin to regulate expression of genes involved in flowering. Additionally, putative AIP1 orthologs are exclusively present in the Angiosperm clade. Furthermore, *AIP1* is mainly expressed in reproductive tissues and plants with reduced expression of *AIP1* show a delay in the timing of flower development. Finally, mRNA levels of genes that are target of ABAP1 or LHP1 transcription repression were down regulated in flower buds of plants with reduced levels of AIP1. Further biochemical and structural analysis of protein complexes containing AIP1/DUF71 will be important to unravel the mechanisms by which Agenet/Tudor integrates these cellular processes.

## Additional files

Additional file 1:
**Schematic representation of the investigated genomes in the plant kingdom for Agenet/Tudor domain proteins and their phylogenetic relationships.** (PDF 552 kb) Additional file 2:
**Modeled structure of Agenet/Tudor domains from plant proteins.** (PDF 925 kb) Additional file 3:
**Temporal expression pattern of each gene from the four different classes of the Agenet/Tudor family.** (PDF 829 kb) Additional file 4:
**AIP1 interaction with ABAP1 domain regions in yeast two-hybrid assays.** (PDF 2041 kb) Additional file 5:
**Analyses of AIP1 interaction with H3K9ac and H3K14ac in pulldown assays.** (PDF 60 kb) Additional file 6:
**Phenotypic analysis of** ***AIP1*** ^***KD***^ **lines during vegetative development.** (PDF 640 kb) Additional file 7:
**All members of Agenet/Tudor family in plants.** (PDF 417 kb) Additional file 8:
**Parameters data from Structural similarity in ESPrint using the second Agenet/Tudor domain from AIP1 protein as query.** (PDF 170 kb) Additional file 9:
**Parameters data from Structural Modeling in I-TASSER.** (PDF 7 kb) Additional file 10:
**List of proteins tested in yeast two hybrid screen using C-terminal portion of AIP1 containing the DUF724 domain as bait.** (PDF 11 kb) Additional file 11:
**Proteins Identified by Tandem Affinity Purification using AIP1 as bait.** (PDF 100 kb) Additional file 12:
**Primers used for PCR cloning and qRT-PCR amplification.** (PDF 10 kb) Additional file 13:
**Supplementary Methods.** (DOCX 26 kb)
