# Supplementary material for: AIP1 is a novel Agenet/Tudor domain protein from Arabidopsis that interacts with regulators of DNA replication, transcription and chromatin remodeling
Source: BMC Plant Biol. 2015 Nov 4;15:270. doi: 10.1186/s12870-015-0641-z (PMC4634149; doi:10.1186/s12870-015-0641-z)
Supplement: Additional file 1: — Schematic representation of the investigated genomes in the plant kingdom for Agenet/Tudor domain proteins and their phylogenetic relationships. (PDF 552 kb) [file 12870_2015_641_MOESM1_ESM.pdf]

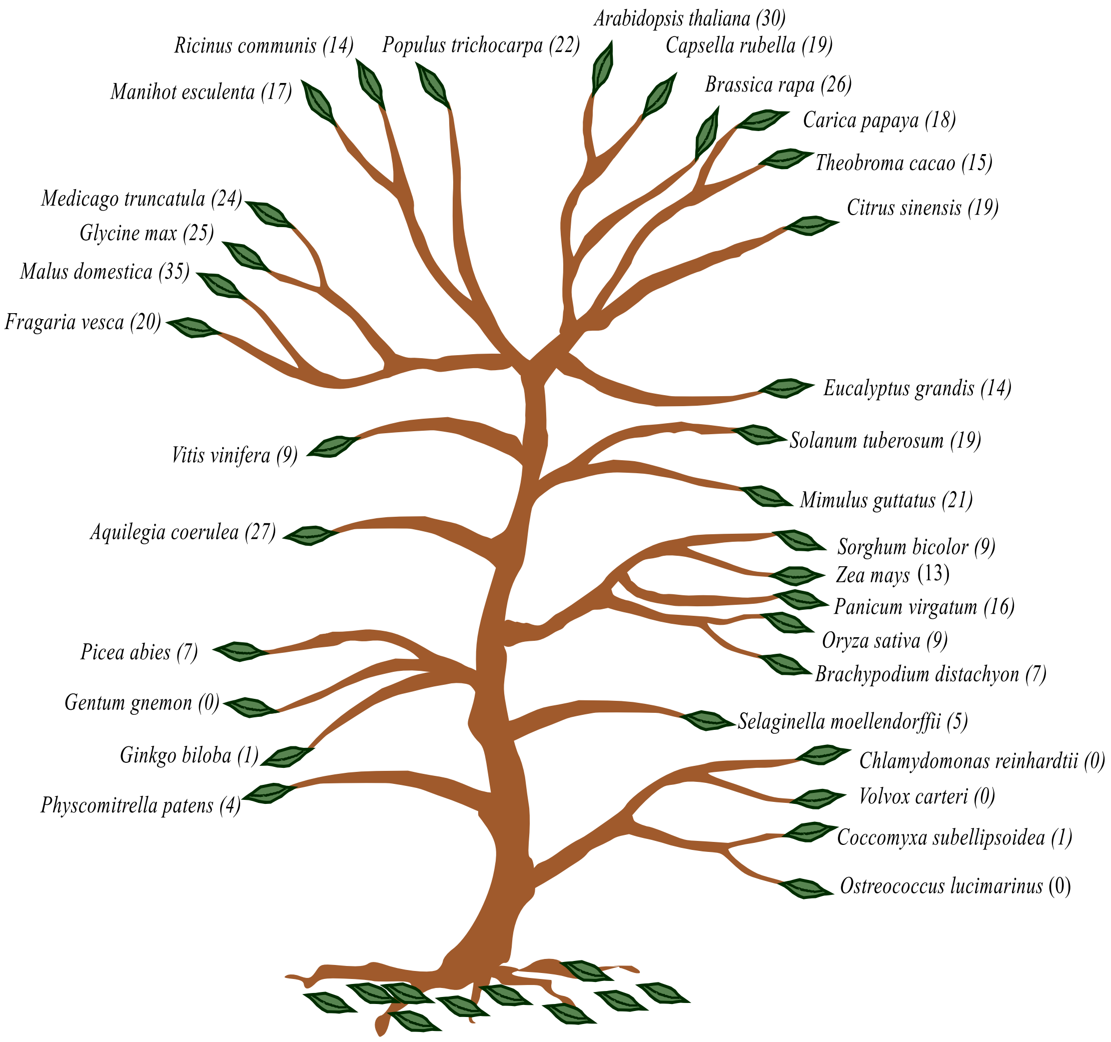

Additional File 1: Schematic representation of the investigated genomes in the plant kingdom for Agenet/Tudor domain proteins and their phylogenetic relationships. The analysis included 446 sequences of 32 species in 24 families from green algae to angiosperms. The values in parenthesis mean the number of proteins containing Agenet/Tudor /Tudor Domain in each species.
