# Supplementary material for: AIP1 is a novel Agenet/Tudor domain protein from Arabidopsis that interacts with regulators of DNA replication, transcription and chromatin remodeling
Source: BMC Plant Biol. 2015 Nov 4;15:270. doi: 10.1186/s12870-015-0641-z (PMC4634149; doi:10.1186/s12870-015-0641-z)
Supplement: Additional file 2: — Modeled structure of Agenet/Tudor domains from plant proteins. (PDF 925 kb) [file 12870_2015_641_MOESM2_ESM.pdf]

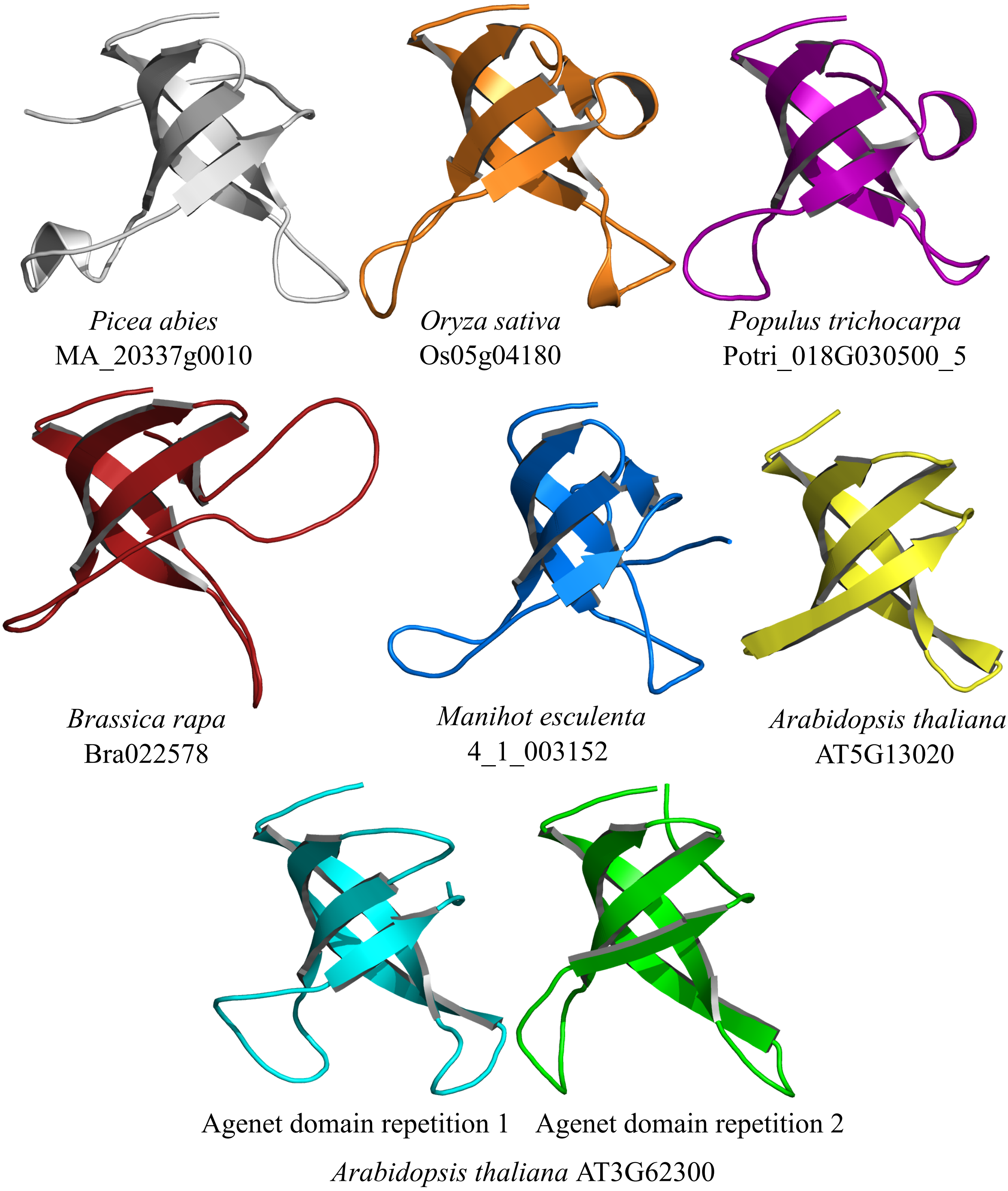

Additional File 2: Modeled structure of Agenet/Tudor domains from plant proteins. The images represent the Agenet/Tudor models generated in the I-TASSER server. The structures are colored in white (B_MA_20337g0010), purple (I_ENT_Potri_018G030500_5), firebrick (I_Central_Bra022578), orange (I_Multiple_Os05g04180), blue (I_BAH_cassava4_1_003152), cyan (D_DUF_AT3G62300.1), yellow (D_DUF_AT5G13020), and green (D_DUF_AT3G62300.2).
