# Supplementary material for: AIP1 is a novel Agenet/Tudor domain protein from Arabidopsis that interacts with regulators of DNA replication, transcription and chromatin remodeling
Source: BMC Plant Biol. 2015 Nov 4;15:270. doi: 10.1186/s12870-015-0641-z (PMC4634149; doi:10.1186/s12870-015-0641-z)
Supplement: Additional file 3: — Temporal expression pattern of each gene from the four different classes of the Agenet/Tudor family. (PDF 829 kb) [file 12870_2015_641_MOESM3_ESM.pdf]

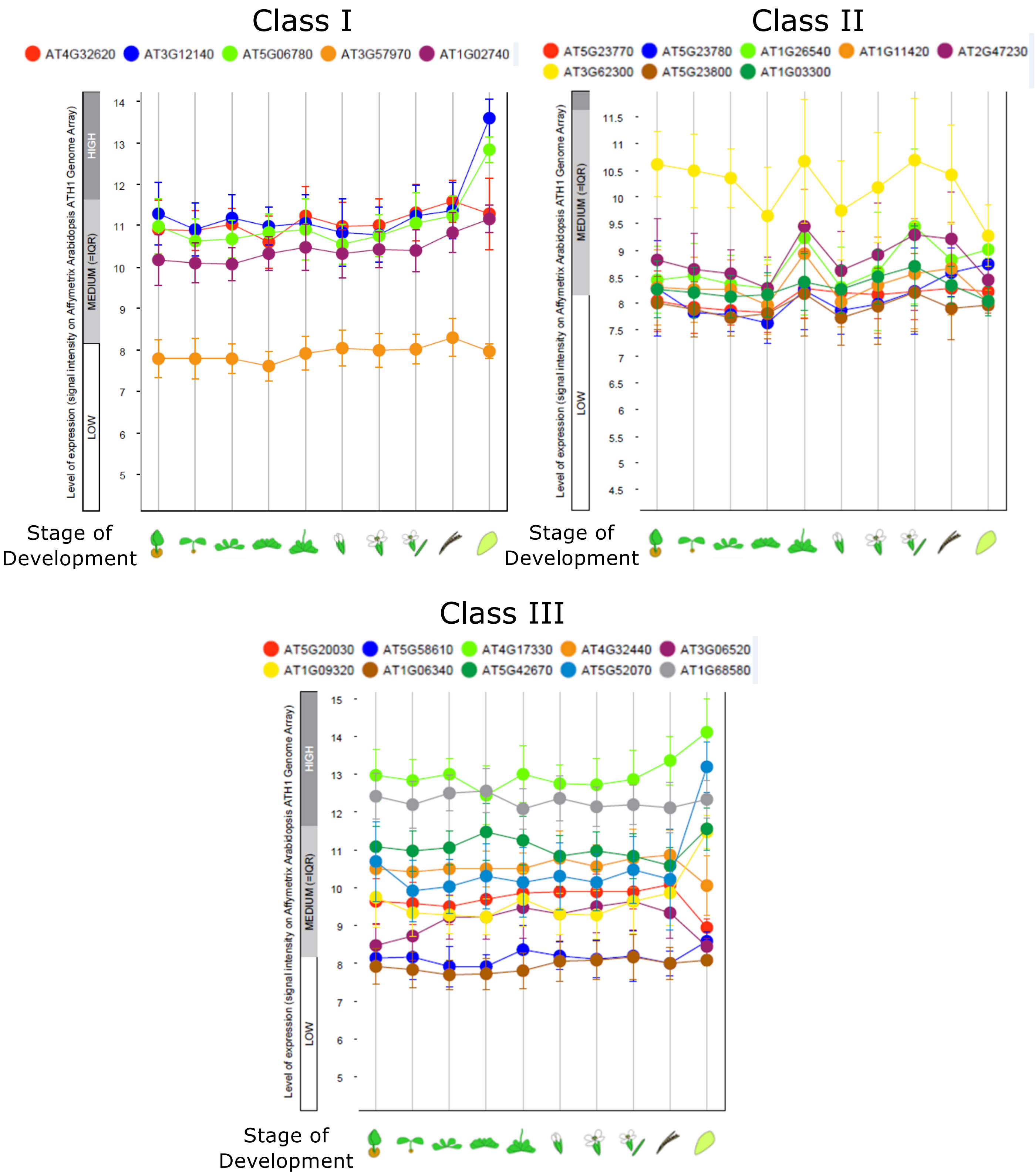

Additional File 3: Temporal expression pattern of each gene from the four different classes of the Agenet/Tudor family. Graphic representation of the average values among the expression values published in many microarray experiments available in Genevestigator. Bars indicate mean ± standard error of different microarray experiments. Each gene is represented in a different color, as specified in the legend. Genes AT5G07350 and AT3G27460 from Class IV are out of analysis since there are no probes in the available microarray data.
