# Supplementary material for: AIP1 is a novel Agenet/Tudor domain protein from Arabidopsis that interacts with regulators of DNA replication, transcription and chromatin remodeling
Source: BMC Plant Biol. 2015 Nov 4;15:270. doi: 10.1186/s12870-015-0641-z (PMC4634149; doi:10.1186/s12870-015-0641-z)
Supplement: Additional file 4: — AIP1 interaction with ABAP1 domain regions in yeast two-hybrid assays. (PDF 2041 kb) [file 12870_2015_641_MOESM4_ESM.pdf]

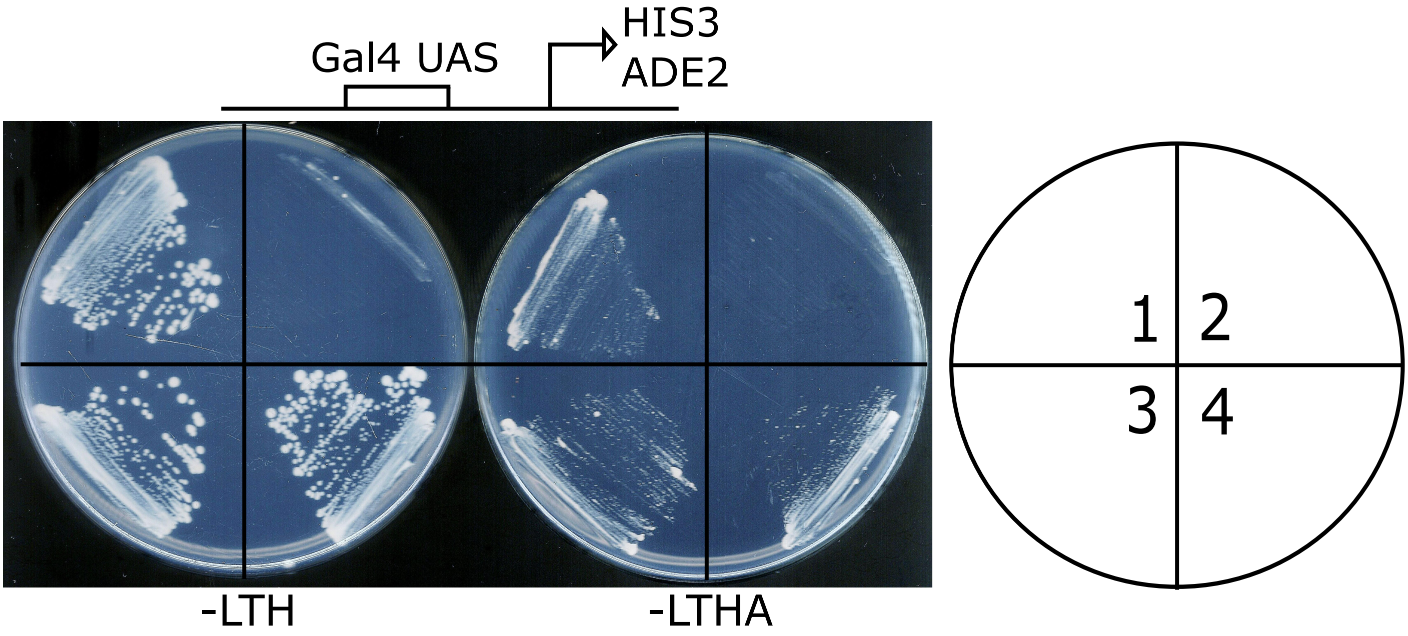


1. AD-DUF724 x BD-ARM
2. AD-DUF724 x BD-BTB
3. AD-DUF724 x BD-ABAP1
4. AD-DUF724 x BD-ARIA

Additional File 4: AIP1 interaction with ABAP1 domain regions in yeast two-hybrid assays. Yeast two hybrid assay with the C-terminal region of AIP1 (aa 540-723 ) fused with GAL4 DAD (AIP1-C-Term AD) against (1) the N-terminal region ABAP1 including the armadillo domains (aa 1-400), or (2) the C-terminal region of ABAP1 containing the BTB-POZ domain (aa 401-737), or (3) the full-length CDS of ABAP1, or (4) the full-length CDS of ARIA, all constructs fused with GAL4 DBD forming the vectors BD-ARM, BD-BTB, BD-ABAP1 or BD-ARIA, respectively. GAL4 DBD empty vector were used as negative control. Details of the constructs can be found in Additional File 1. Yeast transformation was selected in -L-T (SD medium lacking Leucine and Tryptophan), and protein interactions were selected in -L-T-H-A (SD medium lacking Leucine, Tryptophan, Histidine and Adenine). The scheme in the right represents the positions of the yeast co-transformants in the plates.
