# Supplementary material for: AIP1 is a novel Agenet/Tudor domain protein from Arabidopsis that interacts with regulators of DNA replication, transcription and chromatin remodeling
Source: BMC Plant Biol. 2015 Nov 4;15:270. doi: 10.1186/s12870-015-0641-z (PMC4634149; doi:10.1186/s12870-015-0641-z)
Supplement: Additional file 5: — Analyses of AIP1 interaction with H3K9ac and H3K14ac in pulldown assays. (PDF 60 kb) [file 12870_2015_641_MOESM5_ESM.pdf]

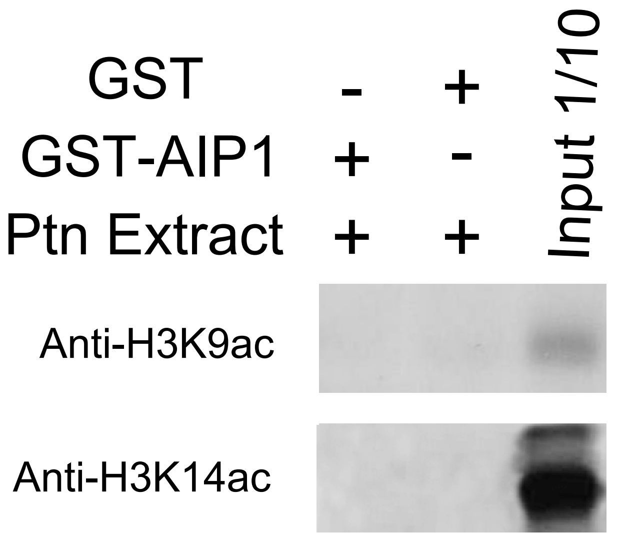

Additional File 5: Analyses of AIP1 interaction with H3K9ac and H3K14ac in pulldown assays. Semi-in vivo pulldown assay of bacterially expressed recombinant GST-AIP1 and protein lysates of Arabidopsis 10-day-old plants. Histone interacting proteins were assayed with anti-H3K9ac and anti-H3K14ac antibodies in immunoblots.
