# Supplementary material for: AIP1 is a novel Agenet/Tudor domain protein from Arabidopsis that interacts with regulators of DNA replication, transcription and chromatin remodeling
Source: BMC Plant Biol. 2015 Nov 4;15:270. doi: 10.1186/s12870-015-0641-z (PMC4634149; doi:10.1186/s12870-015-0641-z)
Supplement: Additional file 6: — Phenotypic analysis of AIP1 KD lines during vegetative development. (PDF 640 kb) [file 12870_2015_641_MOESM6_ESM.pdf]

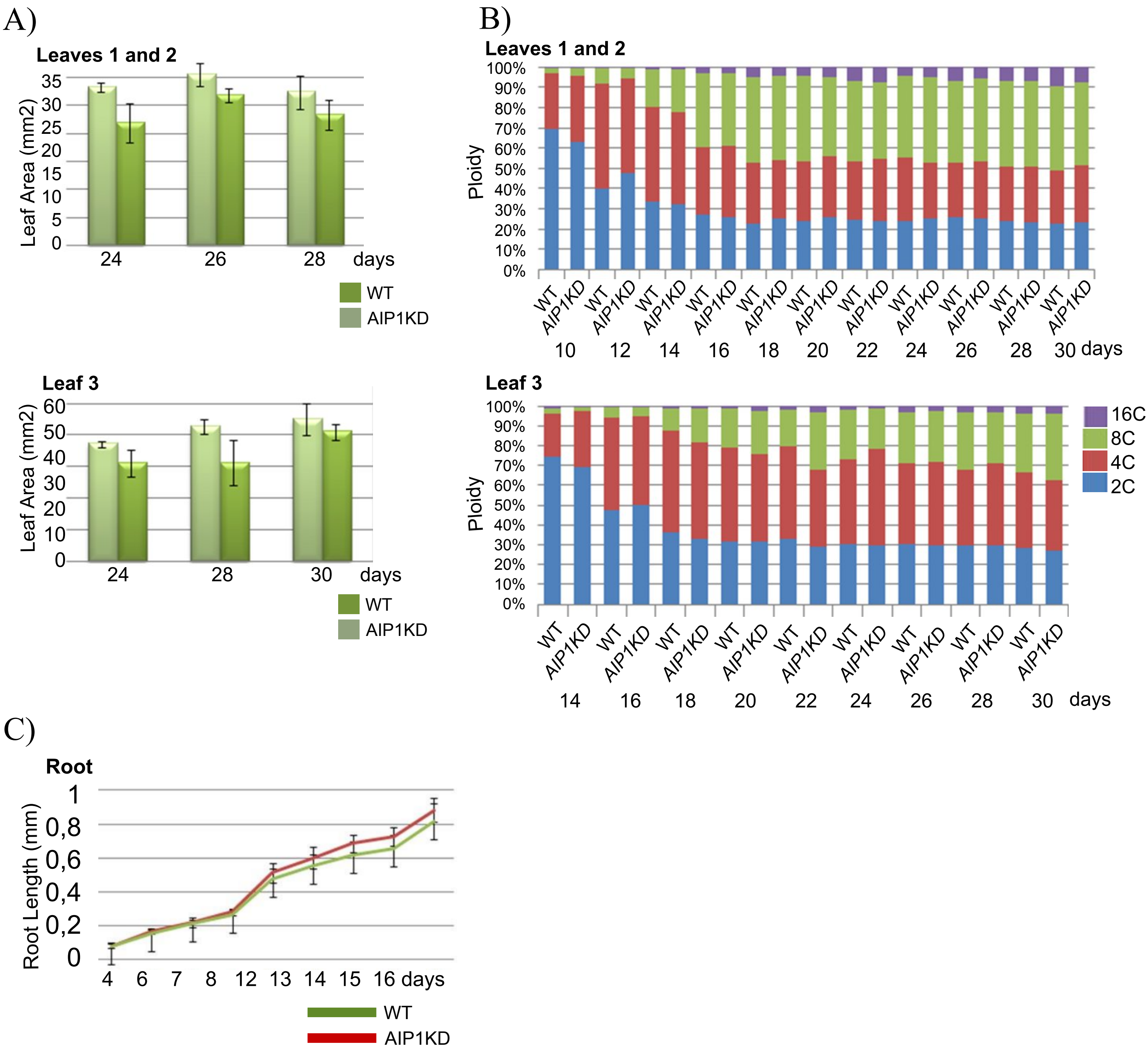

Additional File 6: Phenotypic analysis of AIP1KD lines during vegetative development. (A) Average values of leaves 1-2 (upper panel) and leaf 3 (bottom panel) area measurements in wild-type ecotype Col (darker green) and AIP1KD (lighter green), during development. Absolute values shown are means derived from a pool of 12 plants in 2 independent experiments. Bars indicate mean ± standard error of biological replicates. A statistical analysis was performed by ANOVA (p-value <0.05). In all points analyzed AIP1KD plants are not significantly different from wild type (ANOVA, Po>0.05). (B) The average values of ploidy of cells from leaves 1 and 2 (upper panel) and leaf 3 (bottom panel) of Col-0 WT and AIP1KD plants, harvested in different days along development, were quantified by flow cytometry (See Additional File 1 for details). The values are means derived from a pool of six plants per sample, in 2 independent experiments. The data shows that endoreduplication is not increased in plants with reduced levels of AIP1. (C) Primary root length measurement of wild-type ecotype Col (darker green) and AIP1KD (lighter green) along development. At least six seedlings were grown side by side in 16-8h photoperiod. Absolute values shown are means derived from two independent experiments. Bars indicate mean ± standard error of biological replicates. A statistical analysis was performed by ANOVA (p-value <0.05). All points of the AIP1KD plants were not significantly different from the wild type (ANOVA, Po>0.05). Wild-type (WT), Gabi_645B06 line (AIP1KD), days after germination (days).
