# Supplementary material for: AIP1 is a novel Agenet/Tudor domain protein from Arabidopsis that interacts with regulators of DNA replication, transcription and chromatin remodeling
Source: BMC Plant Biol. 2015 Nov 4;15:270. doi: 10.1186/s12870-015-0641-z (PMC4634149; doi:10.1186/s12870-015-0641-z)
Supplement: Additional file 7: — All members of Agenet/Tudor family in plants. (PDF 417 kb) [file 12870_2015_641_MOESM7_ESM.pdf]

Additional File 7: All members of Agenet/Tudor family in plants. Table revels the locus from 31 genomes where a putative Agenet/Tudor domain were found. It also points out this Agenet/Tudor domain localization in the sequence, and other domain co-existing with it and it´s localization as well. The possibles hothologs in Arabidopsis were mentioned, and AIP1 hortologs are highlighted in yellow.

Green Algae

Ostreococcus lucimarinus

Locus	Agenet/Tudor Domain	Other Domain	Hortolog Ath
No sequence was found			

Coccomyxa subellipsoidea

Locus	Agenet/Tudor Domain	Other Domain	Hortolog Ath
Genemark1.2852_g	Nterm	No	AT5G55600

Volvox carteri

Locus	Agenet/Tudor Domain	Other Domain	Hortolog Ath
No sequence was found			

Chlamydomonas reinhardtii

Locus	Agenet/Tudor Domain	Other Domain	Hortolog Ath
No sequence was found			

Bryophyta

Physcomitrella patens

Locus	Agenet/Tudor Domain	Other Domain	Hortolog Ath
Pp1s94_117V6	Cterm	No	AT4G17330
Pp1s9_69V6	Cterm	No	AT4G17330
Pp1s33_333V6	Cterm	BAH Nterm	AT5G55600
Pp1s235_70V6	Cterm	BAH Nterm	*AT5G55600*

Lycopodiophyta

Selaginella moellendorffii

Locus	Agenet/Tudor Domain	Other Domain	Hortolog Ath
85351	Nterm	No	AT1G06340
405024	Nterm	No	AT5G42670
444321	Central	No	AT4G17330
415212	Cterm	No	AT4G17330
415282	Cterm	BAH Nterm	AT5G55600

Gymnosperm

Ginkgo biloba

Locus	Agenet/Tudor Domain	Other Domain	Hortolog Ath
PgdbGbiloba_1364	Cterm	no	AT4G17330

Gentum gnemon

Locus	Agenet/Tudor Domain	Other Domain	Hortolog Ath
No sequence was found			

Picea abies

Locus	Agenet/Tudor Domain	Other Domain	Hortolog Ath
MA_10134770	Nterm	no	AT1G09320
MA_10425924	Nterm	ENT Cterm	AT1G09320
MA_10119976	Nterm	no	AT1G09320
MA_98820g0010	Nterm	ENT Cterm	AT4G32440
MA_35064g0010	Nterm	ENT Cterm	AT5G20030
MA_20337g0010	Cterm	no	AT4G17330
MA_491766g0010	Nterm	no	AT1G06340

Angiosperm Monocots

Brachypodium distachyon

Locus	Agenet/Tudor Domain	Other Domain	Hortolog Ath
Bradi4g16400	Nterm	No	AT1G09320
Bradi2g38497	Nterm, Central, Cterm	No	AT1G09320
Bradi4g16390	2 x Nterm	DUF724 Cterm	AT2G47230
Bradi4g20140	2 x Nterm	DUF724 Cterm	AT1G09320
Bradi3g07670	Nterm	ENT Cterm	AT5G20030
Bradi3g36500	Cterm	BAH Nterm	AT1G68580
Bradi1g21820	Cterm	BAH Nterm	AT5G55600

Oryza sativa

Locus	Agenet/Tudor Domain	Other Domain	Hortolog Ath
Os06g39900	Nterm	ENT Cterm	AT4G32440
Os02g11000	Nterm	PHD	AT3G14980
Os11g35060	2 x Nterm	DUF724 Cterm	AT3G62300
Os01g37600	2 x Nterm	DUF724 Cterm	AT2G47230
Os05g04180	Nterm, Central, Cterm	No	AT1G09320
Os10g41030	Cterm	No	AT4G17330
Os10g26430	Cterm	BAH Nterm	AT1G68580
Os08g33420	Cterm	BAH Nterm	AT1G68580
Os07g41640	Cterm	BAH Nterm	AT5G55600

Panicum virgatum

Locus	Agenet/Tudor Domain	Other Domain	Hortolog Ath
Pavirv00030603m.g	Nterm	No	AT3G06520
Pavirv00066077m.g	Nterm	PHD Cterm	AT5G58610
Pavirv00010013m.g	Nterm	ENT Cterm	AT4G32440
Pavirv00001885m.g	Nterm	ENT Cterm	AT4G32440
Pavirv00059050m.g	Nterm	DUF724 Cterm	AT3G62300
Pavirv00014438m.g	Nterm	DUF724 Cterm	AT3G62300
Pavirv00018559m.g	2 x Nterm	DUF724 Cterm	AT2G47230
Pavirv00038455m.g	2 x Nterm	DUF724 Cterm	AT2G47230
Pavirv00021402m.g	2 x Nterm	DUF724 Cterm	AT3G62300

Pavirv00058515m.g	Nterm, Central	No	AT1G09320
Pavirv00001452m.g	Nterm, Central	No	AT1G09320
Pavirv00002776m.g	Central	No	AT4G17330
Pavirv00048214m.g	Cterm	BAH Cterm	AT5G55600
Pavirv00057736m.g	Cterm	BAH Cterm	AT1G68580
Pavirv00030084m.g	Cterm	BAH Cterm	AT1G68580
Pavirv00029453m.g	Cterm	BAH Cterm	AT5G55600

Zea mays

Locus	Agenet/Tudor Domain	Other Domain	Hortolog Ath
GRMZM2G160149	Nterm	ENT Cterm	AT4G32440
GRMZM2G048274	2 x Nterm	DUF724 Cterm	AT2G47220
GRMZM2G024641	Nterm, Cterm	No	AT1G09320
GRMZM2G033478	Central, Cterm	No	AT4G17330
GRMZM2G155889	Nterm, Central, Cterm	No	AT1G09320
GRMZM2G340601	Cterm	BAH Nterm	AT1G68580
GRMZM2G037444	Cterm	BAH Nterm	AT5G55600
GRMZM2G419305	Cterm	BAH Nterm	AT5G55600
GRMZM2G336718	Cterm	Start Nterm	AT1G68580
GRMZM2G123644	Cterm	Start and BAH Nterm	AT1G68580
GRMZM2G439819	Cterm	BAH Nterm, Kinase, Rio	AT1G68580
GRMZM2G392624	Central	No	AT1G68580
GRMZM2G049155	Cterm	ENT Nterm	AT5G13020

Sorghum bicolor

Locus	Agenet/Tudor Domain	Other Domain	Hortolog Ath
Sb05g021643	Nterm	No	AT3G06520
Sb05g021640	Nterm	DUF724 Cterm	AT2G47230
Sb03g024845	Nterm	DUF724 Cterm	AT3G06520
Sb02g006590	Nterm	DUF724 Cterm	AT2G47220
Sb03g024845	Nterm	DUF724 Cterm	AT3G06520
Sb10g029020	Nterm	DUF724 Cterm	AT3G62300
Sb01g000510	2 x Nterm	DUF724 Cterm	AT3G62300
Sb07g021050	Cterm	BAH Nterm	AT1G68580
Sb02g038900	Cterm	BAH Nterm	AT5G55600

Angiosperm Eudicot

Aquilegia coerulea

Locus	Agenet/Tudor Domain	Other Domain	Hortolog Ath
AquCa_014_00214	Nterm	No	AT1G06340
AquCa_021_00238	Nterm	No	AT1G06340
AquCa_001_00767	Nterm	No	AT5G20030
AquCa_014_00436	Nterm	No	AT3G06520
AquCa_045_00126	Nterm	No	AT5G58610
AquCa_034_00419	Nterm	No	AT5G42670
AquCa_003_00462	Nterm	WRC Cterm	AT1G09320
AquCa_017_00564	Nterm	ENT Cterm	AT4G32440
AquCa_049_00123	Nterm	ENT Cterm	AT4G32440

AquCa_020_00171	Nterm	ENT Cterm	AT5G20030
AquCa_004_00391	Nterm	PHD Cterm	AT5G58610
AquCa_014_00439	Nterm	UDP-glucosyl transferase	AT3G02100
AquCa_017_00734	Nterm	DUF724	AT3G62300
AquCa_003_00455	Nterm	DUF724	AT1G11420
AquCa_014_00437	Nterm	DUF724	AT3G62300
AquCa_030_00351	Nterm	DUF724	AT3G62300
AquCa_030_00350	Nterm	UF724 Cterm, WRC Nter	AT3G62300
AquCa_030_00352	Nterm	UF724 Cterm, WRC Nter	AT3G62300
AquCa_014_00295	2x Nterm	DUF724	AT3G62300
AquCa_003_00461	2x Nterm	DUF724	AT3G62300
AquCa_003_00458	2x Cterm	UF640 Nterm, WRC Cter	AT5G58500
AquCa_014_00295	2 x Nterm	No	AT3G62300
AquCa_003_00463	Nterm, Cterm	No	AT1G09320
AquCa_013_00777	Cterm	No	AT4G17330
AquCa_002_01142	Cterm	BAH Cterm	AT1G68580
AquCa_018_00032	Cterm	BAH Cterm	AT5G55600
AquCa_110_00042	Cterm	BAH Cterm	AT5G55600

Angiosperm Eudicots Asterid

Mimulus guttatus

Locus	Agenet/Tudor Domain	Other Domain	Hortolog Ath
mgv1a020423m.g	Nterm	PHD Cterm	AT5G58610
mgv1a018116m.g	Nterm	No	AT1G06340
mgv1a026197m.g	Nterm	No	AT2G25590
mgv1a022506m.g	Nterm	No	AT1G09320
mgv1a018116m.g	Nterm	No	AT1G06340
mgv1a009346m.g	Nterm, Cterm	No	AT1G09320
mgv1a005230m.g	Nterm, Central, Cterm	No	AT1G09320
mgv1a005486m.g	Nterm, Central, Cterm	No	AT3G06520
mgv1a001520m.g	2x Nterm	DUF724	AT3G62300
mgv1a000584m.g	Central	No	AT4G17330
mgv1a022930m.g	Central	No	AT5G55600
mgv1a018111m.g	Central	No	AT5G55600
mgv1a022090m.g	Central	No	AT5G55600
mgv1a024218m.g	Central	No	AT5G55600
mgv1a021732m.g	Central	No	AT5G55600
mgv1a022260m.g	Central	No	AT5G55600
mgv1a026054m.g	Central	No	AT5G55600
mgv1a002875m.g	Cterm	BAH Nterm	AT5G55600
mgv1a003408m.g	Cterm	BAH Nterm	AT1G68580
mgv1a021302m.g	Cterm	BAH Nterm	AT1G68580
mgv11b018945m.g	Cterm	No	AT5G55600

Solanum tuberosum

Locus	Agenet/Tudor Domain	Other Domain	Hortolog Ath
PGSC0003DMG402020444	Nterm	No	AT1G06340
PGSC0003DMG400027055	Nterm	No	AT1G06340
PGSC0003DMG400044084	Nterm	No	AT1G09320

PGSC0003DMG400027056	Nterm	No	AT1G06340
PGSC0003DMG400027076	Nterm	No	AT1G06340
PGSC0003DMG400026947	Nterm	No	AT1G06340
PGSC0003DMG400020062	Nterm	No	AT1G06340
PGSC0003DMG400041590	Central	No	AT1G06340
PGSC0003DMG400003974	Central	No	AT5G55600
PGSC0003DMG400035825	Central	No	AT1G06340
PGSC0003DMG400027076	Nterm	No	AT1G06340
PGSC0003DMG400022079	Nterm	PHD Cterm	AT5G58610
PGSC0003DMG400019480	Nterm	ENT Cterm	AT4G32440
PGSC0003DMG400002584	Nterm	DUF724	AT1G11420
PGSC0003DMG400009053	2x Nterm	DUF724	AT2G47230
PGSC0003DMG400015969	2x Nterm, 2x Cterm	No	AT3G06520
PGSC0003DMG400002758	Nterm, Cterm	No	AT3G06520
PGSC0003DMG400027057	Nterm, Cterm	No	AT1G09320
PGSC0003DMG400022180	Cterm	BAH Nterm	AT5G55600

Angiosperm Eudicot

Vitis vinifera

Locus	Agenet/Tudor Domain	Other Domain	Hortolog Ath
GSVIVT01015486001	Nterm	No	AT5G20030
GSVIVT01011913001	Nterm	No	AT1G68580
GSVIVT01035806001	Nterm	ENT Cterm	AT4G32440
GSVIVT01028157001	2 x Nterm	DUF724 Cterm	AT2G47230
GSVIVG01031264001	Central, Cterm	No	AT1G09320
GSVIVT01004716001	Central, Cterm	No	AT4G17330
GSVIVT01003480001	Cterm	BAH Nterm	AT5G55600
GSVIVT01014643001	Cterm	BAH Nterm	AT5G55600
GSVIVT01024715001	Nterm	PHD	AT5G58610

Angiosperm Eudicot Rosid Malvidae

Eucalyptus grandis

Locus	Agenet/Tudor Domain	Other Domain	Hortolog Ath
EuCgr.I00172	Nterm	No	AT5G58610
EuCgr.B01689	Nterm	No	AT1G09320
EuCgr.J01526	Nterm	No	AT1G06340
EuCgr.H00912	Nterm	DUF724 Cterm	AT2G47230
EuCgr.C04172	Nterm	ENT Cterm	AT4G32440
EuCgr.C01115	Nterm	ENT Cterm	AT4G32440
EuCgr.J01892	Nterm	PHD Cterm	AT5G58610
EuCgr.L00253	Nterm, Cterm	No	AT1G09320
EuCgr.H04076	Nterm, Cterm	No	AT1G09320
EuCgr.E01742	Nterm, Cterm	No	AT1G09320
EuCgr.D01444	Cterm	BAH Nterm	AT5G55600
EuCgr.F03695	Cterm	BAH Nterm	AT5G55600
EuCgr.B03027	Cterm	BAH Nterm	AT1G68580
EuCgr.D02089	Cterm	No	AT5G55600

Citrus sinensis

Locus	Agenet/Tudor Domain	Other Domain	Hortolog Ath
oraNge1.1g038506m.g	Nterm	No	AT1G06340
oraNge1.1g047914m.g	Nterm	No	AT1G06340
oraNge1.1g046641m.g	Nterm	No	AT4G32440
oraNge1.1g000791m.g	Nterm	PHD Cterm	AT5G58610
oraNge1.1g004709m.g	Cterm	BAH Nterm	AT1G68580
oraNge1.1g008969m.g	Cterm	BAH Nterm	AT1G68580
oraNge1.1g044464m.g	Cterm	BAH Nterm	AT5G55600
oraNge1.1g014914m.g	Nterm	ENT Cterm	AT5G20030
oraNge1.1g012860m.g	Nterm	ENT Cterm	AT5G20030
oraNge1.1g014912m.g	Nterm	ENT Cterm	AT5G20030
oraNge1.1g012860m.g	Nterm	ENT Cterm	AT5G20030
oraNge1.1g016251m.g	Nterm	ENT Cterm	AT4G32440
oraNge1.1g019582m.g	Nterm, Cterm	No	AT1G09320
oraNge1.1g019553m.g	Nterm, Cterm	No	AT1G09320
oraNge1.1g002548m.g	2x Nterm	DUF724 Cterm	AT3G62300
oraNge1.1g000115m.g	Cterm	No	AT4G17330
oraNge1.1g004709m.g	Cterm	BAH Nterm	AT1G68580
oraNge1.1g044464m.g	Cterm	BAH Nterm	AT5G55600
oraNge1.1g006118m.g	Cterm	BAH Nterm	AT5G55600

Theobroma cacao

Locus	Agenet/Tudor Domain	Other Domain	Hortolog Ath
TheCC1EG021952	Nterm	No	AT1G06340
TheCC1EG029700	Nterm	No	AT1G06340
TheCC1EG028359	Nterm	No	AT2G25590
TheCC1EG001536	Nterm	No	AT5G58610
TheCC1EG020809	Nterm	No	AT5G42670
TheCC1EG038002	Nterm	ENT Cterm	AT5G20030
TheCC1EG041594	Nterm	PHD Cterm	AT5G58610
TheCC1EG019808	Nterm	DUF724 Cterm	AT2G47230
TheCC1EG004869	2x Nterm	DUF724 Cterm	AT2G47230
TheCC1EG015949	Cterm	No	AT4G17330
TheCC1EG030410	Cterm	BAH Nterm	AT5G55600
TheCC1EG011826	Cterm	BAH Nterm	AT1G68580
TheCC1EG030669	Cterm	BAH Nterm	AT5G55600
TheCC1EG026790	Nterm, Cterm	No	AT1G09320
TheCC1EG021425	Nterm, Central, Cterm	No	AT3G06520

Carica papaya

Locus	Agenet/Tudor Domain	Other Domain	Hortolog Ath
evm.TU.superCoNtig_18.95	Nterm	No	AT1G11420
evm.TU.CoNtig_31482	Nterm	No	AT3G62300
evm.TU.superCoNtig_52.171	Nterm	No	AT1G06340
evm.TU.superCoNtig_52.173	Nterm	No	AT1G06340
evm.TU.superCoNtig_693	Nterm	No	AT1G06340
evm.TU.CoNtig_45528	Nterm	No	AT1G06340
evm.TU.CoNtig_34571	Nterm	No	AT1G09320

evm.TU.superCoNtig_551	Nterm	No	AT1G06340
evm.TU.superCoNtig_75.67	Nterm	No	AT5G20030
evm.TU.superCoNtig_52.170	Nterm	No	AT1G06340
evm.TU.superCoNtig_693	Nterm	No	AT1G06340
evm.TU.superCoNtig_29.172	Cterm	No	AT4G17330
evm.TU.superCoNtig_551	Nterm	No	AT1G06340
evm.TU.CoNtig_43820	Nterm	No	AT1G06340
evm.TU.superCoNtig_427	Nterm	ENT Cterm	AT4G32440
evm.TU.superCoNtig_48.181	Nterm, Cterm	No	AT1G09320
evm.TU.superCoNtig_75.67	Nterm	No	AT5G20030
evm.TU.superCoNtig_52.172	Central	No	AT1G06340

Brassica rapa

Locus	Agenet/Tudor Domain	Other Domain	Hortolog Ath
Bra022578	Nterm	No	AT5G42670
Bra029141	Nterm	No	AT5G42670
Bra032427	Nterm	No	AT1G06340
Bra038440	Nterm	No	AT2G47230
Bra003495	Nterm	;ylation Nterm, DUF724 (	AT3G62300
Bra020080	Nterm	ENT Cterm	AT5G20030
Bra011348	Nterm	T Central, PPR repeat Ct	AT2G25580
Bra040078	Nterm	ENT Cterm	AT4G32440
Bra004469	Nterm	DUF724 Cterm	AT2G47230
Bra036420	Nterm	DUF724 Cterm	AT1G11420
Bra004468	Nterm	DUF724 Cterm	AT2G47230
Bra040222	Nterm	DUF724 Cterm	AT2G47230
Bra020753	2x Nterm	DUF724 Cterm	AT2G47230
Bra040223	2x Nterm	DUF724 Cterm	AT2G47230
Bra003496	2x Nterm	DUF724 Cterm	AT3G62300
Bra014402	2x Nterm	DUF724 Cterm	AT3G62300
Bra033167	2x Nterm	DUF724 Cterm	AT3G62300
Bra007670	2x Nterm	DUF724 Cterm	AT3G62300
Bra038871	2x Nterm	DUF724 Cterm	AT1G11420
Bra017883	Nterm, Central	No	AT1G11420
Bra031661	Nterm, Central, Cterm	No	AT1G09320
Bra018567	Nterm, Central, Cterm	No	AT1G09320
Bra028969	Cterm	BAH Nterm	AT5G55600
Bra002890	Cterm	BAH Nterm	AT5G55600
Bra004029	Cterm	BAH Nterm	AT1G68580
Bra004322	Cterm	BAH Nterm	AT1G68580

Capsella rubella

Locus	Agenet/Tudor Domain	Other Domain	Hortolog Ath
Carubv10002476m.g	Nterm	ENT Cterm	AT5G20030
Carubv10025786m.g	Nterm	PHD Cterm	AT5G58610
Carubv10023445m.g	Nterm	ENT Cterm	AT4G32440
Carubv10006237m.g	Nterm	ENT Cterm	AT4G32440
Carubv10011095m.g	Nterm	No	AT1G06340
Carubv10000478m.g	Nterm	DUF724 Cterm	AT5G23770

Carubv10016739m.g	2x Nterm	DUF724 Cterm	AT3G62300
Carubv10012522m.g	2x Nterm	DUF724 Cterm	AT1G03300
Carubv10010977m.g	2x Nterm	DUF724 Cterm	AT1G26540
Carubv10000562m.g	2x Nterm	DUF724 Cterm	AT5G23800
Carubv10000545m.g	2x Nterm	DUF724 Cterm	AT5G23800
Carubv10022757m.g	2x Nterm	DUF724 Cterm	AT2G47230
Carubv10013669m.g	2x Nterm, Central, Cterm	No	AT3G06520
Carubv10008882m.g	Nterm, Central, Cterm	No	AT1G09320
Carubv10003965m.g	Cterm	No	AT4G17330
Carubv10020075m.g	Cterm	BAH Nterm	AT1G68580
Carubv10020073m.g	Cterm	BAH Nterm	AT1G68580
Carubv10019941m.g	Cterm	BAH Nterm	AT1G68580
Carubv10026036m.g	Cterm	BAH Nterm	AT5G55600

Angiosperm Eudicot Rosid Fabidae

Fragaria vesca

Locus	Agenet/Tudor Domain	Other Domain	Hortolog Ath
geNe00235.1-v1.0-hybrid	Nterm	No	AT1G09320
geNe05025.1-v1.0-hybrid	Nterm, Cterm	No	AT3G06520
geNe18196.1-v1.0-hybrid	Nterm	No	AT1G06340
geNe20316.1-v1.0-hybrid	Nterm	No	AT1G06340
geNe22310.1-v1.0-hybrid	Nterm	No	AT1G06340
geNe20318.1-v1.0-hybrid	Nterm	No	AT1G06340
geNe30975.1-v1.0-hybrid	Nterm	ENT Cterm	AT4G32440
geNe20274.1-v1.0-hybrid	Nterm	ENT Cterm	AT5G20030
geNe11691.1-v1.0-hybrid	Nterm	ENT Cterm	AT4G32440
geNe07936.1-v1.0-hybrid	Nterm	PHD Cterm	AT5G58610
geNe20576.1-v1.0-hybrid	Nterm, Cterm	DUF724 Cterm	AT2G47230
geNe21408.1-v1.0-hybrid	Nterm, Cterm	DUF724 Cterm	AT3G62300
geNe21409.1-v1.0-hybrid	Nterm	DUF724 Cterm	AT3G62300
geNe12406.1-v1.0-hybrid	Nterm, Cterm	No	AT1G09320
geNe19813.1-v1.0-hybrid	Cterm	BAH Nterm	AT5G55600
geNe03763.1-v1.0-hybrid	Cterm	BAH Nterm	AT1G68580
geNe23526.1-v1.0-hybrid	Cterm	WLM Cterm, BAH Nterm	AT5G55600
geNe08916.1-v1.0-hybrid	Cterm	No	AT4G17330
geNe12404.1-v1.0-hybrid	Cterm	7x PPR repeat	AT5G42310
geNe23370.1-v1.0-hybrid	Nterm	No	AT1G09320

Malus domestica

Locus	Agenet/Tudor Domain	Other Domain	Hortolog Ath
MDP0000122350	Nterm	No	AT1G09320
MDP0000278262	Nterm	No	AT1G09320
MDP0000320348	Nterm	No	AT1G09320
MDP0000148323	Nterm	No	AT1G09320
MDP0000169032	Nterm	No	AT1G06340
MDP0000169202	Nterm	No	AT1G06340
MDP0000273144	Nterm	No	AT1G56320
MDP0000688188	Nterm	No	AT1G06340

MDP0000235367	Nterm	No	AT1G68580
MDP0000274363	Nterm	No	AT1G26540
MDP0000138467	Nterm, Cterm	No	AT1G09320
MDP0000203214	2x Nterm	No	AT1G26540
MDP0000138467	Nterm, Cterm	No	AT1G09320
MDP0000322413	Nterm, Cterm	No	AT1G09320
MDP0000242277	Nterm	se, Response regulator C	AT2G47430
MDP0000281445	Nterm	F-box Nterm, DNaJ Cterm	AT5G49580
MDP0000126098	Nterm	ENT Cterm	AT5G20030
MDP0000250177	Nterm	PPR repeat, DUF260 Cte	AT3G10572
MDP0000183528	Nterm	DUF724 Cterm	AT2G47230
MDP0000193434	Nterm	DUF724 Cterm	AT2G47230
MDP0000212039	Nterm	DUF724 Cterm	AT3G62300
MDP0000122929	2x Nterm	DUF724 Cterm	AT2G47230
MDP0000139988	2x Nterm	DUF724 Cterm	AT2G47230
MDP0000244230	Central	PRMT5 Cterm, ENT Cterm	AT1G04870
MDP0000304352	Central	ling lectin Nterm, ENT Ct	AT4G32440
MDP0000143447	Cterm	BAH Nterm	AT1G68580
MDP0000245750	Cterm	BAH Nterm	AT1G68580
MDP0000163443	Cterm	BAH Nterm	AT5G55600
MDP0000604089	Cterm	BAH Nterm	AT1G68580
MDP0000282378	Cterm	BAH Nterm	AT5G55600
MDP0000231374	Cterm	BAH Nterm	AT5G55600
MDP0000312564	Cterm	BAH Nterm	AT5G55600
MDP0000319854	Cterm	BAH Nterm	AT5G55600
MDP0000245314	Cterm	BAH Nterm	AT5G55600
MDP0000262281	Cterm	No	AT4G17330

Glycine max

Locus	Agenet/Tudor Domain	Other Domain	Hortolog Ath
Glyma05g04731	Cterm	No	AT4G17330
Glyma12g00251	Nterm	No	AT5G42670
Glyma06g43070	Nterm	No	AT1G06340
Glyma12g15170	Nterm	No	AT1G06340
Glyma06g07391	Nterm	No	AT4G32440
Glyma05g08291	Nterm	No	AT5G20030
Glyma09g26255	Nterm	No	AT1G09320
Glyma04g07330	Nterm	ENT Cterm	AT4G32440
Glyma17g12693	Nterm	ENT Cterm	AT5G20030
Glyma02g07171	Nterm	F-box Nterm	AT1G09320
Glyma16g29630	Nterm	F-box Nterm	AT4G10400
Glyma02g39300	Nterm	PHD Cterm	AT5G58610
Glyma11g27622	Nterm	PHD Cterm	AT5G58610
Glyma11g27510	Nterm	PHD Cterm	AT5G58610
Glyma10g28671	Central	No	AT1G09320
Glyma09g03740	Nterm, Cterm	No	AT1G09320
Glyma20g22810	Nterm, Cterm	No	AT1G09320
Glyma01g41721	Cterm	No	AT4G17330
Glyma11g03650	Cterm	No	AT4G17330

Glyma0022s00420	Cterm	BAH Nterm	AT1G68580
Glyma01g20775	Cterm	BAH Nterm	AT1G68580
Glyma18g08850	Cterm	BAH Nterm	AT5G55600
Glyma08g43990	Cterm	BAH Nterm	AT5G55600
Glyma15g12410	Cterm	BAH Nterm	AT5G55600
Glyma09g01511	Cterm	BAH Nterm	AT5G55600

Medicago truncatula

Locus	Agenet/Tudor Domain	Other Domain	Hortolog Ath
Medtr2g033390	Nterm, Central	No	AT3G06520
Medtr4g079830	Nterm	No	AT1G06340
Medtr5g089140	Nterm	No	AT1G06340
Medtr5g089220	Nterm	No	AT1G06340
Medtr5g089250	Nterm	No	AT1G06340
Medtr4g079980	Nterm	No	AT1G06340
Medtr3g065560	Nterm	No	AT1G06340
Medtr4g027030	Nterm	No	AT5G58610
Medtr1g019680	Nterm	No	AT1G06340
Medtr4g127390	Nterm	No	AT1G06340
AC233684_4	Nterm	No	AT1G09320
Medtr4g024780	Nterm	F-box Nterm	AT3G58980
Medtr4g023710	Nterm	F-box Nterm	AT1G09320
Medtr7g017280	Nterm	F-box Nterm	AT3G58980
Medtr4g026550	Nterm	F-box Nterm	AT1G09320
Medtr4g113670	Nterm	ENT Cterm	AT5G20030
Medtr3g107680	Nterm	DUF724 Cterm	AT4G32440
Medtr5g071340	Nterm	PDH	AT5G58610
Medtr4g024810	Nterm	NB-ARC Cterm	AT1G09320
Medtr2g026390	Cterm	BAH Nterm	AT5G55600
Medtr1g084560	Central	No	AT1G09320
Medtr5g012780	Cterm	No	AT4G17330
Medtr4g100800	Cterm	No	AT4G17330
Medtr5g055210	Cterm	BAH Nterm	AT1G68580

Populus trichocarpa

Locus	Agenet/Tudor Domain	Other Domain	Hortolog Ath
Potri.001G364300	Cterm	BAH Nterm	AT5G55600
Potri.009G151200	Nterm	No	AT1G06340
Potri.008G119400	Nterm	No	AT1G68580
Potri.001G163500	Nterm	No	AT3G62300
Potri.004G193200	Nterm	No	AT1G06340
Potri.002G118600	Nterm	PDH	AT5G58610
Potri.005G216200	Nterm	PDH	AT5G20030
Potri.009G072600	Nterm	PDH	AT5G58610
Potri.006G250600	Nterm	ENT Cterm	AT4G32440
Potri.018G030500	Nterm	ENT Cterm	AT4G32440
Potri.002G046800	Nterm	ENT Cterm	AT4G32440
Potri.002G192300	2 x Nterm	DUF724 Cterm	AT2G47230
Potri.014G117800	2 x Nterm	DUF724 Cterm	AT2G47230

Potri.005G011100	Nterm, Cterm	No	AT1G09320
Potri.013G006900	Nterm, Cterm	No	AT1G09320
Potri.010G149300	Nterm, Central	No	AT3G06520
Potri.006G017400	Cterm	BAH Nterm	AT5G55600
Potri.010G126700	Cterm	BAH Nterm	AT1G68580
Potri.001G365800	Cterm	BAH Nterm	AT5G55600
Potri.016G007500	Cterm	BAH Nterm	AT5G55600
Potri.001G157300	Cterm	No	AT4G17330
Potri.003G077600	Cterm	No	AT4G17330

Ricinus communis

Locus	Agenet/Tudor Domain	Other Domain	Hortolog Ath
29900.t000084	Nterm	No	AT1G06340
57892.t000001	Nterm	No	AT1G09320
57944.t000001	Nterm	No	AT1G09320
30128.t000003	Nterm	No	AT2G47230
29900.t000085	Nterm	No	AT1G06340
29883.t000033	Nterm	ENT Cterm	AT4G32440
29841.t000059	Nterm	ENT Cterm	AT4G32440
30208.t000006	2 x Nterm	DUF724 Cterm	AT2G47230
30128.t000393	Nterm, Cterm	No	AT1G09320
29889.t000112	Nterm, Central, Cterm	No	AT3G06520
30101.t000002	Cterm	No	AT4G17330
29629.t000008	Cterm	BAH Nterm	AT5G55600
28883.t000009	Cterm	BAH Nterm	AT1G68580
30076.t000178	Nterm	AT-hook Cterm	AT3G62300

Manihot esculenta

Locus	Agenet/Tudor Domain	Other Domain	Hortolog Ath
Cassava4.1_010435m	Nterm, Cterm	No	AT3G06520
Cassava4.1_024563m	Nterm, Cterm	No	AT3G06520
Cassava4.1_003739m	2 x Nterm	No	AT3G06520
Cassava4.1_003561m	2 x Nterm	No	AT1G11420
Cassava4.1_004413m	Nterm	No	AT2G47230
Cassava4.1_021242m	Nterm	No	AT1G06340
Cassava4.1_009683m	Nterm	ENT Cterm	AT4G32440
Cassava4.1_003152m	Cterm	BAH Nterm	AT5G55600
Cassava4.1_002945m	Cterm	BAH Nterm	AT5G55600
Cassava4.1_011107m	Nterm	No	AT2G25590
Cassava4.1_029040m	Nterm	PDH	AT5G20030
Cassava4.1_002893m	Cterm	BAH Nterm	AT1G68580
Cassava4.1_032472m	Nterm	ENT Cterm	AT5G20030
Cassava4.1_000049m	Cterm	No	AT4G17330
Cassava4.1_000055m	Cterm	No	AT4G17330
Cassava4.1_010435m	Nterm, Cterm	No	AT1G09320
Cassava4.1_010763m	Nterm	No	AT5G42670

Arabidopsis thaliana

Locus	Agenet/Tudor Domain	Other Domain

AT5G42670	Nterm	No
AT5G52070	Nterm	No
AT4G32440	Nterm	No
AT1G06340	Nterm	No
AT1G09320	Nterm, Cterm	No
AT3G06520	Nterm 2x, Cterm 2x	No
AT5G20030	Nterm	ENT Cterm
AT5G58610	Nterm	PHDCterm
AT5G23770DUF8	Nterm	DUF724 Cterm
AT5G23780DUF9	Nterm	DUF724 Cterm
AT1G03300DUF1	Nterm 2x	DUF724 Cterm
AT1G26540DUF3	Nterm 2x	DUF724 Cterm
AT1G11420DUF2	Nterm 2x	DUF724 Cterm
AT2G47230DUF6	Nterm 2x	DUF724 Cterm
AT3G62300DUF7	Nterm 2x	DUF724 Cterm
AT5G23800DUF10	Nterm 2x	DUF724 Cterm
AT3G12140	Cterm	ENT Nterm
AT3G57970	Cterm	ENT Nterm
AT5G06780	Cterm	ENT Nterm
AT2G25590	Cterm	BAH Nterm
AT5G55600	Cterm	BAH Nterm
AT1G68580	Cterm	BAH Nterm
AT4G17330	Cterm	No
AT5G40550	Tudor Cterm	No
AT3G27460	Tudor Cterm	No
AT2G02570	Tudor Central	No
AT4G32620	Tudor Nterm	No
AT1G02740	Tudor Nterm	MRG Cterm
AT5G61780	Tudor Cterm	Snase 5x N and Cterm
AT5G07350	Tudor Cterm	Snase 4x N and Cterm
