# Supplementary material for: AIP1 is a novel Agenet/Tudor domain protein from Arabidopsis that interacts with regulators of DNA replication, transcription and chromatin remodeling
Source: BMC Plant Biol. 2015 Nov 4;15:270. doi: 10.1186/s12870-015-0641-z (PMC4634149; doi:10.1186/s12870-015-0641-z)
Supplement: Additional file 8: — Parameters data from Structural similarity in ESPrint using the second Agenet/Tudor domain from AIP1 protein as query. (PDF 170 kb) [file 12870_2015_641_MOESM8_ESM.pdf]

Additional File 8: Parameters data from Structural similarity in ESPrint using the second Agenet/Tudor domain from AIP1 protein as query. The *z* -score indicates overall model quality (Figure 1a). Its value is displayed in a plot that contains the *z* -scores of all experimentally determined protein chains in current PDB. The higher Z-score, the higher is statistical significance of the match. No. SSE is the number of matched secondary structure elements (SSEs) – i.e. helices and strands - between the two structures. RMSD is the the root mean square deviation (RMSD) between the C-alpha atoms of the matched residues in the best 3D superposition of the query and target structures. Seq. Id for amino acid sequence identity between the two proteins. PDB for entry codes in Protein DataBase.

Hit	Z-score	No. SSE	RMSD (Å)	Seq. id	PDB
1	8.2	4	1.2	22.4%	4hczA
2	8.5	4	1.39	23.1%	3qiiA
3	7.7	4	1.33	21.8%	3pmtA
4	8.3	4	1.34	19.0%	4bd3A
5	6.6	4	1.48	17.3%	3s6wA
6	6.9	3	1.5	13.8%	4qq6A
7	6.4	3	1.51	22.4%	2ltoA
8	6.4	3	1.23	19.0%	2e5qA
9	8.4	4	1.17	22.6%	3p8dA
