# Supplementary material for: AIP1 is a novel Agenet/Tudor domain protein from Arabidopsis that interacts with regulators of DNA replication, transcription and chromatin remodeling
Source: BMC Plant Biol. 2015 Nov 4;15:270. doi: 10.1186/s12870-015-0641-z (PMC4634149; doi:10.1186/s12870-015-0641-z)
Supplement: Additional file 9: — Parameters data from Structural Modeling in I-TASSER. (PDF 7 kb) [file 12870_2015_641_MOESM9_ESM.pdf]

Additional File 9: Parameters data from Structural Modeling in I-TASSER. Scoring function (C-score) is based on the relative clustering structural density and the consensus significance score of multiple threading templates to estimate the accuracy of the I-TASSER predictions. Numbers can vary from -5 to 2 and higher numbers are more significant. TM-score is a measure of the absolute distance between atoms of the final model in comparison with the native structure. Ideal TM-Score is higher than 0,5.

**Gene**	**C--score**	**TM--score**
MA_20337g0010	-1.20	0.56±0.15
Potri_018G030500_5	-0.51	0.65±0.13
Bra022578	-1.08	0.58±0.14
Os05g04180	-0.46	0.65±0.13
cassava4_1_003152	-1.71	0.51±0.15
AT3G62300.1	-1.18	0.57±0.15
AT5G13020	0.02	0.72±0.11
AT3G62300.2	-0.82	0.61±0.14
