# Supplementary material for: AIP1 is a novel Agenet/Tudor domain protein from Arabidopsis that interacts with regulators of DNA replication, transcription and chromatin remodeling
Source: BMC Plant Biol. 2015 Nov 4;15:270. doi: 10.1186/s12870-015-0641-z (PMC4634149; doi:10.1186/s12870-015-0641-z)
Supplement: Additional file 10: — List of proteins tested in yeast two hybrid screen using C-terminal portion of AIP1 containing the DUF724 domain as bait. (PDF 11 kb) [file 12870_2015_641_MOESM10_ESM.pdf]

Additional File 10: List of proteins tested in yeast two hybrid screen using C-terminal portion of AIP1 containing the DUF724 domain as bait. 2HD, for yeast two hybrid. P, for positive interactions and N, for negative.

Gene	Function	2HD
ABAP1	Cell cycle control	P
ARM	N-Term portion of ABAP1	P
BTB	C-Term portion of ABAP1	N
ARIA	Interacts with ABAP1	P
TCP24	Interacts with ABAP1	N
ORC1a	Pre-RC member	N
ORC2	Pre-RC member	N
ORC4	Pre-RC member	N
ORC5	Pre-RC member	N
ORC6	Pre-RC member	N
CDT1a	Pre-RC member	N
CDT1b	Pre-RC member	N
CDC6	Pre-RC member	N
MCM2	Pre-RC member	N
MCM3	Pre-RC member	N
MCM7	Pre-RC member	N
HBO1	Histone Acetylase	N
HDAC1	Histone Acetylase	N
HDAC2	Histone Acetylase	N
DRB4	Histone Acetylase	N
LHP1	Interacts with methylated histone	P
H4	Histone 4	N
SNF2/RAD54	Helicase-Like	N
PP2a	Phosphatase	N
DBB	DNA Repair	N
