# Supplementary material for: AIP1 is a novel Agenet/Tudor domain protein from Arabidopsis that interacts with regulators of DNA replication, transcription and chromatin remodeling
Source: BMC Plant Biol. 2015 Nov 4;15:270. doi: 10.1186/s12870-015-0641-z (PMC4634149; doi:10.1186/s12870-015-0641-z)
Supplement: Additional file 11: — Proteins Identified by Tandem Affinity Purification using AIP1 as bait. (PDF 100 kb) [file 12870_2015_641_MOESM11_ESM.pdf]

Additional File 11: Proteins Identified by Tandem Affinity Purification Using AIP1 as bait. Three trials of the same experiment were done. In the third trial, a small peptide with a conserved sequence of the Histone Superfamily was rescued, but mass spectrometry could not determine the specific histone (s) that interact with AIP1, in between eight possible candidates. Besides histones, two other proteins were suggested to bind to AIP1.

Accession N°	Protein name	N-terminal tag fusion, trial 1	N-terminal tag fusion, trial 2	N-terminal tag fusion, trial 3	Total*
AT3G62300	AIP1	1	1	1	3
AT1G76810	eIF-2 family protein	0	1	1	2
AT1G60770	TPR-like superfamily protein	0	0	1	1
AT1G07790;	Histone superfamily protein	0	0	1	1
AT2G28720;	Histone superfamily protein				
AT2G37470;	Histone superfamily protein				
AT3G45980;	Histone superfamily protein				
AT3G46030;	Histone superfamily protein				
AT5G02570;	Histone superfamily protein				
AT5G22880;	Histone superfamily protein				
AT5G59910	Histone superfamily protein				

*Total from three experiments.
