# Supplementary material for: AIP1 is a novel Agenet/Tudor domain protein from Arabidopsis that interacts with regulators of DNA replication, transcription and chromatin remodeling
Source: BMC Plant Biol. 2015 Nov 4;15:270. doi: 10.1186/s12870-015-0641-z (PMC4634149; doi:10.1186/s12870-015-0641-z)
Supplement: Additional file 12: — Primers used for PCR cloning and qRT-PCR amplification. (PDF 10 kb) [file 12870_2015_641_MOESM12_ESM.pdf]

Additional File 12: Primers used for PCR cloning and qRT-PCR amplification.

Primer name	Sequence(5´-3´)
AIP1attb1F	GGGGACAAGTTTGTACAAAAAAGCAGGCTGAAAGCATGTTGTTAACTACGGGAA
AIP1attb2R	GGGGACCACTTTGTACAAGAAAGCTGGGTCTTATTTCCATGGGGCAGC
ABAP1attb1F	ATGATAATCTCAAAATCGTTCA
ABAP1attb2R	TTATACGACGGTTGGGGG
ARIAattb1	ATGGACCAACAACCGGAG
ARIAattb2	CAACCTCAAGCTTTGCAGGT
LHP1attb1	ATGAAAGGGGCAAGTGGTG
LHP1attb2	AGGCGTTCGATTGTACTTGAG
qRT-PCR AIP1 F	AGCTCGGATGCAGTCTCAAGCAG
qRT-PCR AIP1 R	TGGGGCAGCGAGAATTGCCTG
qRT-PCR UBI14 F	TCACTGGAAAGACCATTACTCTTGAA
qRT-PCR UBI14 R	AGCTGTTTTCCAGCGAAGATG
qRT PCR UBI10 F	CACACTCCACTTGGTCTTGCGCT
qRT PCR UBI10 R	TGGTCTTTCCGGTGAGGAGTCTTCA
qRT PCR GAPDH F	TTGGTGACAACAGGTCAAGCA
qRT PCR GAPDH R	AAACTTGTCGCTCAATGCAATC
qRT PCR CyclinB1;1 F	CCTCCATTCACTCTCAACAG
qRT PCR CyclinB1;1 R	CCTGGCAGCTGTGGAATATG
qRT PCR CDKB2;1 F	TGTTCTTGCCAGTGGCTACGG
qRT PCR CDKB2;1 R	CACGTGGTCAGGTTAATGGA
qRT PCR CDT1b F	AAATGTCGACTGCCGAAACAG
qRT PCR CDT1b R	AAGTGAAATGTCATGTGAAGTTGCTT
qRT PCR PCNA2 F	ATCTGGCGCCTAAGATTGAA
qRT PCR PCNA2 R	CAACAAAGGACTCGAACGAA
qRT PCR FT F	CCAAGTCCTAGCAACCCTCA
qRT PCR FT R	TACACTGTTTGCCTGCCAAG
qRT PCR AG F	CAAAACTCCAACAGGCAATTG
qRT PCR AG R	CATTTTCAGCTATCTTTGCAC
qRT PCR AP3 F	GCCCTAACACCACAACGAAGG
qRT PCR AP3 R	CTCACCTAGCCTCTGCTTGATC
