# Supplementary material for: AIP1 is a novel Agenet/Tudor domain protein from Arabidopsis that interacts with regulators of DNA replication, transcription and chromatin remodeling
Source: BMC Plant Biol. 2015 Nov 4;15:270. doi: 10.1186/s12870-015-0641-z (PMC4634149; doi:10.1186/s12870-015-0641-z)
Supplement: Additional file 13: — Supplementary Methods. (DOCX 26 kb) [file 12870_2015_641_MOESM13_ESM.docx]

**Supplementary Methods**

**Constructs**

The full length coding regions of AIP1, ABAP1, LHP1 and ARIA, and the C-terminus of AIP1 (amino acids positions 540 to 722) were amplified by PCR with the primers indicated in the Additional File 13. The constructs were generated using BP and LR reactions of the Gateway® Technology (Life Technologies). The amplified PCR fragments were cloned in pDONR221. The entry clones were transferred to the following destination vectors: (1) the bacteria expression vectors pDEST15 for GST fusion and pDEST17 for His fusion; (2) to the yeast two‑hybrid vectors pGWAD for Gal4 activation domain fusion and pGWBD for Gal4 binding domain fusion (Life Technologies); (3) to the 35S::GFP fusion vector (pK7WGF2) and the 35S::RFP fusion vector (pH7WGR2) for subcellular localization. For TAP assays, the full length CDS of AIP1 was cloned in NGSrhino destination vector. For yeast two hydrid assays, the constructs with full length sequences of ABAP1 and ARIA, and the N-terminus and C- terminus of ABAP1 (called ARM and BTB) were previously reported in Masuda et. al (2008).

***In silico* analyses of proteins containing Agenet/Tudor domain**

Sequences used to bild Agenet/Tudor signiture in plant via WebLogo: GRMZM2G160149; GSVIVT01024715001; PGSC0003DMG400027056; Pp1s9_69V6; Sm444321; Cpa_supercontig_75_67; MDP0000169032; MDP0000278262; MDP0000212039; MDP0000126098; MDP0000498356; MA_20337g0010; MA_98820g0010; Carubv10011095m; Thecc1EG021952; Glyma11g27510; Glyma05g04731; Sb03g024845; Sb05g021640; Medtr1g019680; Medtr7g017280; Os10g26430; Os11g35060.1; Os11g35060.2; Bradi4g20140.2; Bradi4g20140.1; Mdo20274; Mdo20316; cassava4_1_024563.1; cassava4_1_024563.2; orange1_1g002548m.2; orange1_1g047914m; orange1_1g002548m.1; mgv1a005486m.1; mgv1a020423m; Bra040078; Bra020753.2; Bra020753.1; Rco29841_m002795; Rco57892_t000001; Rco28883_m000719; Aquca_030_00351_2; Aquca_021_00238; Aquca_034_00419; Pavirv00021402m.1; Pavirv00021402m.2; mgv1a001520m; mgv1a022506m; mgv1a005486m.2; Potri_001G163500; Potri_002G046800; Potri_002G046800; Eeucgr_B01689; Eucgr_J01526.

**Molecular and phenotypic analysis of AIP1 mutant plants**

Insertion lines of GABI_645B06 were identified by genotyping, according to Gabi-Kat methods (https://www.gabi-kat.de/). Briefly, DNA was isolated from one rosette leaf of 20 day-old plants. Genotypes of thirty plants were determined by PCR with specific primers for GABI T-DNA insertion and for AIP1. For genotyping, primers o8409 LB 5’- LP ATATTGACCATCATACTCATTGC -3’ and AIP1 specific LP 5'-TTAATCCAGCAGTACCCGATG -3' and RP 5'-GGTTTCAGGTAGCGAGGAAAG-3' were used to identify WT, heterozygous or homozygous lines for the mutation.

For phenotypic analysis of GABI and wild type plants, the methodology of kinematics was used to analyze leaf growth, according to De Veylder et al (2001) and Fiorani and Beemster (2006). Plants were harvested from day 6 until day 21 after sowing for microscopy. Plants were mounted on a slide and covered. The leaf primordia was observed under a microscope fitted with differential interference contrast optics (DMLB; Leica, Wetzlar, Germany). The total (blade) area of leaves 1 and 2 of each plant was determined from drawing-tube images with the public domain image analysis program ImageJ (version 1.30; http://rsb.info.nih.gov/ij/) (Erickson, 1976).

The development of roots was accompanied during days 4 to 16 after germination, according to Cazzonelli et al. (2013). Measurements were carried out of the length of main roots of plants cultivated in plates in vertical position, with growth medium (1.5% agar supplemented with modified Hoagland solution, 1% sucrose, 0.5% MES; pH = 5.7).

For phenotyping reproductive structures, 30 day-old plants were used to count the number of leaves, flower buds, developed flowers (open flowers) and siliques. To evaluate the timing of flower development by counting the number of flower buds per inflorescence, the first inflorescences of plants 22-24 day-old were harvested and dissected when their first flower opened.

Flow cytometry analyses were done essentially as described in Boudolf et al. (2004).

**Expression analyses**

Total RNA was extracted from the frozen material according to Logemann et al. (1987). To eliminate the residual genomic DNA present in the preparation, the RNA was treated by RNAse-free DNAse I according to the manufacturer’s instructions (Amersham Biosciences). Total RNA was quantified with nanodrop and loaded onto an agarose gel to check its integrity. First strand cDNA was synthesized using “First Strand cDNA Taqman Kit” (Applied Biosystems) with oligo (dT) primer solution and 0.5 ug RNA template, according to the manufacturer’s instructions. The oligonucleotides used for qRT‑PCR were designed in gene‑specific regions with primer Express 2.0 (Perkin Elmer Applied Biosystems, Foster City, CA) or Primer3 software and are listed in Additional File 12. The cDNA was amplified using SYBR-Green® PCR Master kit (Perkin-Elmer Applied Biosystem) in the GeneAmp 9600 thermocycler (Perkin-Elmer Applied Biosystems) under standard conditions. *AtUBI10* and *GAPDH* constitutive genes were used as cDNA amount control for data presented in Figure 10; and *AtUBI14* was used for data presented in Figure 9. Data were calculated using the mathematical formula 2^[CTubi14-CTgene]^ and were further normalized to the level of the wild type controls.

***In vitro* and semi-*in vivo* protein interaction assays**

AIP1-GST and ABAP1-HIS, ARIA-HIS, LHP1-HIS and AIP1-HIS were produced in cells of *E. coli* strain BL21 as described by Chekanova et al (2000), with modification in the lysis buffer (25 mM Tris, pH 8.0, 1 mM EDTA, 10% glycerol, 50 mM NaCl, 0.1% Triton X‑100, 1 mM phenylmethylsulfonyl fluoride (PMSF), 10 mM leupeptin, and 75 mM aprotinin). GST pulldown analyses were carried out according to Tarun & Sachs (1996).

For the semi-*in vivo* pulldown experiments, proteins were extracted from 10 day-old seedlings, by grounding to a fine powder in liquid nitrogen, followed by extraction in 50-100µL of extraction buffer (5% ethylene glycol; 25 mM Tris-HCl pH 7.6; 15 mM MgCl_2;_ 5 mM EGTA pH 8; 150 mM NaCl; 15 mM para-nitrophenylphosphate; 60 mM beta-glycerophosphate; 1 mM dithiothreitol; 0.1% NP-40; 0.1 mM Na3VO4}; 1 mM NaF; 1 mM PMSF; 10 µg/ml leupeptin; 10 µg/ml aprotinin; 10 µg/ml soybean trypsin inhibitor; 0.1 mM benzamidin; 5 µg/ml antipain; 5 µg/ml pepstatin; 5 µg/ml chymostatin; 1 µM E64). The extracts were centrifuged at 20,000 × g for 10 min, and the supernatant was incubated as performed in the *in vitro* pulldown assays.

For protein gel blot, proteins were separated by 10% SDS‑PAGE and blotted onto Immobilion‑P membranes (Millipore, Bedford, MA). Membranes were blocked (5% milk powder, 150 mM NaCl, 0.05% Tween 20, 25 mM Tris‑Cl, pH 8.0) for 2h at room temperature and incubated for 1h with antibodies against ABAP1 (1:1000; Covance Corp.), HIS (1:1000, Sigma, St.Louis, MO), GST (1:5000; Sigma, St.Louis, MO) or HISTONES (1:2000; Santa Cruz Biotechnology) in blocking buffer. Detection was carried out according to the ECL Western Blotting System according to manufacturer´s instructions (GE‑Healthcare).

Anti-ABAP1 polyclonal antibody was developed against the peptide antigen GAPIVTQLID (amino acids 28 to 37), by Covance Corp (Masuda et al., 2008). The following antibodies were used: anti-histone H1 (Santa Cruz H1 antibody AF-4, SC8030); anti-H2B (Santa Cruz H2B antibody FL126, SC10808); anti-H3 (Santa Cruz H3 antibody **FL136, C10809); anti-H4 (Santa Cruz H4 antibody H-97, SC10810), anti- H3K9ac** (Milipore , 07352); anti- H3K14ac (Milipore,07353).

**Supplementary References**

Boudolf V, Vlieghe K, Beemster GTS, Magyar Z, Acosta JAT, Maes S, et al. The plant-specific Cyclin-Dependent Kinase CDKB1;1 and transcription factor E2Fa-DPa control the balance of mitotically dividing and endoreduplicating cells in Arabidopsis. Plant Cell. 2004;16:2683-92.

Cazzonelli CI, Vanstraelen M, Simon S, Yin K, Carron-Arthur A, Nisar N. Role of the Arabidopsis PIN6 Auxin Transporter in Auxin Homeostasis and Auxin-Mediated Development. Plos One. 2013;8(7):e70069.

Chekanova JA, Shaw RJ, Wills MA, Belostotsky DA. Poly(A) tail‑dependent exonuclease AtRrp41p from A. thalianarescues 5.8 S rRNA processing and mRNA decay defects of the yeast ski6 mutant and is found in an exosome‑sized complex in plant and yeast cells. J Biol Chem. 2000;275:33158-33166.

De Veylder L, Beeckman T, Beemster GTS, Krols L, Terras F, Landrieu I. Functional Analysis of Cyclin-Dependent Kinase Inhibitors of Arabidopsis. The Plant Cell. 2001;13(7):1653-1668.

Erickson, R.O.  Modeling of plant growth. Annu. Rev. Plant Physiol. 1976;27:407-434.

[Fiorani F](http://www.ncbi.nlm.nih.gov/pubmed/?term=Fiorani%20F%5BAuthor%5D&cauthor=true&cauthor_uid=16724264), [Beemster GT](http://www.ncbi.nlm.nih.gov/pubmed/?term=Beemster%20GT%5BAuthor%5D&cauthor=true&cauthor_uid=16724264). Quantitative analyses of cell division in plants. [Plant Mol Biol.](http://www.ncbi.nlm.nih.gov/pubmed/16724264) 2006;60;6:963-79.

Logemann, J, Schell J, Willmitzer L. Improved Method for the Isolation of RNA from Plant Tissues. Analytical Biochem. 1987;163:16-20.

Masuda HP, Cabral LM, De Veylder L, Tanurdzic M, Engler JD, Geelen D, Inze D, Martienssen RA, Ferreira PCG. Hemerly AS. ABAP1 is a novel plant Armadillo BTB protein involved in DNA replication and transcription. EMBO J. 2008;27:2746-2756.

Tarun SZ, Sachs AB. Association of the yeast poly(A) tail binding protein with translation initiation factor eIF-4G. EMBO J. 1996;15:7168-7177.
